# Effect of Frequency of Changing Point-of-Use Reminder Signs on Health Care Worker Hand Hygiene Adherence: A Cluster Randomized Clinical Trial

**DOI:** 10.1001/jamanetworkopen.2019.13823

**Published:** 2019-10-23

**Authors:** Mark W. Vander Weg, Eli N. Perencevich, Amy M. J. O’Shea, Michael P. Jones, Mary S. Vaughan Sarrazin, Carrie L. Franciscus, Cassie Cunningham Goedken, Gio J. Baracco, Suzanne F. Bradley, Jose Cadena, Graeme N. Forrest, Kalpana Gupta, Daniel J. Morgan, Michael A. Rubin, Joseph Thurn, Marvin J. Bittner, Heather Schacht Reisinger

**Affiliations:** 1 Center for Access and Delivery Research and Evaluation, Iowa City VA Health Care System, Iowa City, Iowa; 2 Department of Internal Medicine, University of Iowa, Iowa City; 3 Department of Psychological and Brain Sciences, University of Iowa, Iowa City; 4 Department of Epidemiology, University of Iowa, Iowa City; 5 Department of Biostatistics, University of Iowa, Iowa City; 6 Miami VA Healthcare System, Miami, Florida; 7 VA Ann Arbor Healthcare System, Ann Arbor, Michigan; 8 South Texas Veterans Health Care System, San Antonio; 9 Department of Medicine, University of Texas Health Science Center at San Antonio, San Antonio; 10 VA Portland Health Care System, Portland, Oregon; 11 VA Boston Healthcare System, Boston, Massachusetts; 12 VA Maryland Health Care System, Baltimore; 13 VA Salt Lake City Health Care System, Salt Lake City, Utah; 14 Minneapolis VA Medical Center, Minneapolis, Minnesota; 15 Nebraska-Western Iowa Veterans Affairs Health Care System, Omaha, Nebraska

## Abstract

**Importance:**

Although hand hygiene (HH) is considered the most effective strategy for preventing hospital-acquired infections, HH adherence rates remain poor.

**Objective:**

To examine whether the frequency of changing reminder signs affects HH adherence among health care workers.

**Design, Setting, and Participants:**

This cluster randomized clinical trial in 9 US Department of Veterans Affairs acute care hospitals randomly assigned 58 inpatient units to 1 of 3 schedules for changing signs designed to promote HH adherence among health care workers: (1) no change; (2) weekly; and (3) monthly. Hand hygiene rates among health care workers were documented at entry and exit to patient rooms during the baseline period from October 1, 2014, to March 31, 2015, of normal signage and throughout the intervention period of June 8, 2015, to December 28, 2015. Data analyses were conducted in April 2018.

**Interventions:**

Hospital units were randomly assigned into 3 groups: (1) no sign changes throughout the intervention period, (2) signs changed weekly, and (3) signs changed monthly.

**Main Outcomes and Measures:**

Hand hygiene adherence as measured by covert observation. Interrupted time series analysis was used to examine changes in HH adherence from baseline through the intervention period by group.

**Results:**

Among 58 inpatient units, 19 units were assigned to the no change group, 19 units were assigned to the weekly change group, and 20 units were assigned to the monthly change group. During the baseline period, 9755 HH opportunities were observed at room entry and 10 095 HH opportunities were observed at room exit. During the intervention period, a total of 15 855 HH opportunities were observed at room entry, and 16 360 HH opportunities were observed at room exit. Overall HH adherence did not change from baseline compared with the intervention period at either room entry (4770 HH events [48.9%] vs 3057 HH events [50.1%]; *P* = .14) or exit (6439 HH events [63.8%] vs 4087 HH events [65.2%]; *P* = .06). In units that changed signs weekly, HH adherence declined from baseline at room entry (−1.9% [95% CI, −2.7% to −0.8%] per week; *P* < .001) and exit (−0.8% [95% CI, −1.5% to 0.1%] per week; *P* = .02). No significant changes in HH adherence were observed in other groups.

**Conclusions and Relevance:**

The frequency of changing reminder signs had no effect on HH rates overall. Units assigned to change signs most frequently demonstrated worsening adherence. Considering the abundance of signs in the acute care environment, the frequency of changing signs did not appear to provide a strong enough cue by itself to promote behavioral change.

**Trial Registration:**

ClinicalTrials.gov Identifier: NCT02223455

## Key Points

**Question:**

Does changing reminder signs more frequently in a hospital setting improve health care workers’ hand hygiene adherence?

**Findings:**

In this cluster randomized clinical trial of 58 inpatient units in 9 acute care hospitals, overall hand hygiene adherence did not change significantly between before and during the intervention period at patient room entry or exit. In units assigned to change signs most frequently, hand hygiene decreased at patient room entry and exit.

**Meaning:**

Hand hygiene adherence in the hospital setting is not likely to be affected solely by more frequent changes of reminder signs.

## Teaser

This cluster randomized trial investigates the effect of frequency of changing hand hygiene reminder signs on hand hygiene adherence among health care workers.

## Introduction

Direct contact during patient care is a primary means of transmitting hospital-acquired infections (HAIs).^1,2^ Consequently, proper hand hygiene (HH) is considered the most effective strategy for reducing incidence of HAIs.^3,4^ Despite recognition of the importance of HH to patient care, adherence rates tend to be low. A 2010 systematic review^5^ of studies of adherence with HH guidelines in the hospital environment reported a mean adherence rate of 40%. Thus, there is significant room for improving HH practices to prevent HAI.

Studies on the effect of HH initiatives on HAI rates have been hampered by methodological limitations, including lack of adequate control groups, small numbers of observations, short observation periods, and insufficient attention to theory.^6^ However, the available evidence suggests that improving HH can reduce infection rates.^7,8^ Visual reminders, such as signs, are a relatively inexpensive and frequently used strategy for promoting HH adherence. Because they are commonly implemented as part of a multimodal intervention strategy or bundle, the independent effect of reminder signs has been difficult to discern. Although results have been mixed,^9,10,11^ limited evidence indicates that visual cues can be an effective strategy for increasing HH rates, particularly if the cues incorporate messages informed by health communication theory.^12,13^ However, to our knowledge, little else is known about how to design and implement visual reminders to maximize their effectiveness.

Although not exhaustively studied in the context of health care reminders, findings from the literature on characteristics of effective warnings may be applicable. For example, a 1993 study^14^ suggested that the conspicuousness of warnings appeared to affect awareness, recall, and effectiveness. According to the Communication-Human Information Processing model,^15^ the ability to be noticed and attended to are the essential first requirements of an effective warning. Therefore, designing cues with features that enable them to be detected within the noise of the information overload that characterizes the clinical environment is crucial.

Another factor that is associated with the effectiveness of visual cues is habituation, which refers to the tendency for a response to a stimulus to diminish after repeated exposures. This process of adaptation appears to have a neural basis, as neuroimaging and electroencephalography studies, such as a 2006 study by Grill-Spector et al,^16^ have demonstrated reduced activity in brain regions involved in information processing after repeated presentations of a stimulus. Consequently, signs that are encountered regularly may eventually cease to serve as an effective cue to action and fail to generate the desired response (eg, hand cleaning). Therefore, even well-designed signs may lose their impact on HH adherence over time.

One strategy for reducing habituation is to change a reminder’s stimulus properties so that it continues to attract attention.^17,18,19^ Findings on characteristics of effective warning messages suggest that changing the appearance of a message can facilitate attention by enhancing conspicuity or salience, thereby reducing habituation.^18,19^ A 2015 functional magnetic resonance imaging study^20^ suggested that warning signs that change in appearance were more resistant to habituation as measured by activity in visual processing regions of the brain. However, to our knowledge, little attention has been paid to how frequently signs should be changed to reduce health care worker habituation and maintain high levels of HH adherence.

The primary aim of this study was to evaluate the effect of changing reminder signs on health care worker HH adherence. Accordingly, outcomes were assessed and analyzed at the level of individual patient encounters during patient room entry and exit. We hypothesized that regularly changing signs would increase their ability to be noticed and protect against habituation, resulting in improved HH adherence rates. The project was part of an ongoing study to identify combinations of strategies to optimize HH adherence in the acute care setting.

## Methods

This study was approved by the Department of Veteran’s Affairs Office of Research and Development Central Institutional Review Board. Since the project posed no more than minimal risk, the intervention consisted of an environmental manipulation (frequency of changes in signage) that targeted all health care workers on the units, outcomes focused on routine health care worker behavior, and no patient-level data were obtained, individual-level informed consent was not required from either patients or health care workers. The study was conducted in accordance with the Consolidated Standards of Reporting Trials (CONSORT) reporting guideline extension for cluster randomized trials.^21^

### Study Design

This study used a cluster randomized clinical design. Because the intervention occurred at the population level and involved making changes to the patient care environment, individual random assignment at the patient or health care worker level was not feasible. Inpatient medical units were sorted and randomized in blocks of 3 according to preintervention adherence rates to 3 different interventions. Random assignment was conducted by our study biostatistician (M.P.J.) using a computerized randomly generated number sequence. Nineteen units were randomly assigned to keep the same signs throughout the intervention period (no change group), 19 units were assigned to change them on a weekly basis (weekly group), and 20 units were assigned to change them every month (monthly group). Because the intervention targeted health care worker behavior through publicly visible cues (ie, signs), blinding to unit assignment of clinical staff and those assessing outcomes was not attempted. From October 1, 2014, to March 31, 2015, baseline HH adherence data were collected with existing signage conditions. No effort was made to standardize baseline signage across units or sites. Beginning on June 8, 2015, economic and health behavior change theory–informed signs were placed next to or on the alcohol-based hand sanitizer (ABHS) dispenser located at the entry to patient rooms. Signs were subsequently changed according to each unit’s assigned schedule. Individual signs included pictures of different health care workers and patients and used a variety of color schemes but were otherwise identical in content (eAppendix in Supplement 1). Observations for the intervention period continued until December 28, 2015, based on an a priori designated duration of approximately 6 months. Primary analyses were performed in April 2018. The full trial protocol is available in Supplement 2.

### Setting

**Figure 1.**
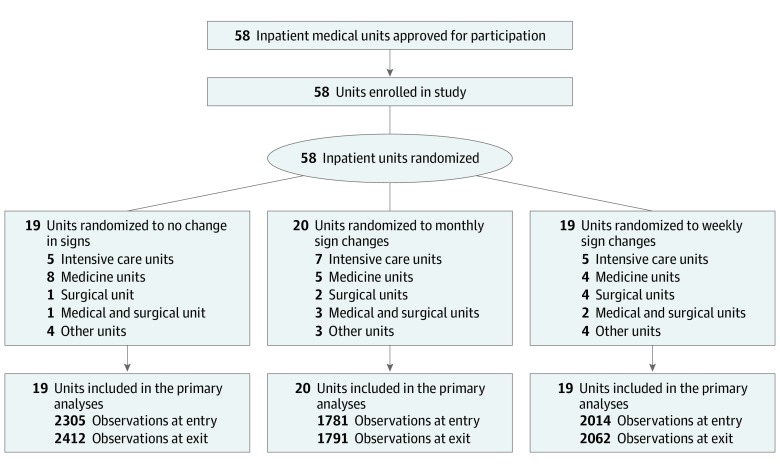
CONSORT Diagram of Unit Recruitment and Randomization

This study was set in 58 units at 9 US Department of Veterans Affairs acute care hospitals participating in a larger collaborative effort focused on reducing methicillin-resistant *Staphylococcus aureus* infections and other HAIs (Figure 1). Sites were chosen to represent diversity in geographic location and hospital size. This study was conducted on all general medical wards, surgical wards, intensive care units, bone marrow transplant units, and spinal cord units at each medical center. Psychiatric units were excluded owing to restrictions on ABHS in patient care areas. Information regarding overall observation activity is included in the eTable in Supplement 1.

### Intervention: Reminder Signs

Hand hygiene reminder signs were placed next to or directly on ABHS dispensers. In most units, the signs were positioned by the door outside of individual patient rooms. The signs measured 21.59 cm by 27.94 cm at 2 hospitals, 13.97 cm by 10.79 cm at 6 hospitals, and 7.62 cm by 10.16 cm at 1 hospital. The signs and their physical locations were designed to serve as visual cues to prompt health care worker HH at the point of room entry and exit. All health care workers engaged in patient care on study units were considered targets of the intervention and contributed to study outcomes.

Sign content was grounded in communication and health behavior theories, including prospect theory^22^ and message framing,^23^ which posit that the way a message is framed is likely to influence decision-making. Health communication messages can be designed to emphasize either the benefits (ie, gain-framed) or losses (ie, loss-framed) associated with a given behavior or its absence. A 1997 review by Rothman and Salovey^23^ suggested that gain-framed messages would be more persuasive for encouraging prevention behaviors associated with little risk (eg, hand washing). Conversely, detection behaviors, such as cancer screening, which are characterized by the potential for risk (ie, identifying the presence of disease), are likely to be affected more by messages emphasizing loss. Consistent with this view, prior work has shown that gain-framed messages that focused on positive outcomes associated with proper HH (as opposed to those that emphasized avoiding negative consequences) and on the outcomes for patients rather than on health care workers themselves are associated with better HH adherence.^12,13,24^ Consequently, all intervention reminder signs incorporated these characteristics.

### Primary Outcomes

Hand hygiene adherence was determined by direct covert observation following procedures we have used in prior studies.^24,25^ Observers, who were not health care workers on the study units, were stationed in the hallway and documented HH performance at patient room entry and exit. Observations were recorded on a standardized data collection form which included site, unit, room, date, time, HH adherence, method (ie, soap and water, wall-mounted ABHS, or personal ABHS), and profession of the observed health care worker. Observers were provided with a cover story to mask the true reason for their presence. Based on prior work indicating that HH adherence tends to increase after observers have been on the unit for approximately 15 minutes,^26^ which may reflect reactivity to being monitored, observation periods were limited to 15 minutes in an effort to reduce the so-called Hawthorne effect. Observations were conducted primarily on weekdays during the day shift, although there was some variability across sites. To ensure adequate surveillance across units, sites provided a list of all room numbers for each of their units through which observers were instructed to sequentially cycle over time.

### Sample Size

Initial sample size calculations indicated that 30 000 observations in each group would result in 99.0% power to detect a 5.0% increase in hand hygiene rates due to the intervention (eg, 60.0% to 65.0%). However, because initial power calculations did not account for observation clustering within units, sample size requirements were recalculated after baseline data were collected so that a reliable estimate of the intraclass correlation coefficient could be obtained. Based on an intraclass correlation coefficient of 0.07 and assuming baseline HH adherence rates of 45.0% (room entry) and 63.0% (room exit), an estimated 250 observations per unit were determined to be needed to achieve a power of 0.80 at a Bonferroni-corrected type I error rate of 0.017.

### Statistical Analysis

Entry and exit HH adherence were calculated for units in each treatment group. Crude (unadjusted) change in HH adherence from baseline to follow-up was assessed for each intervention group using Fisher exact test. For the primary analyses, HH adherence before and after implementing the intervention was investigated using interrupted time series analysis with autoregressive error models to account for autocorrelation and trends before and during the intervention period for entry and exit overall, as well as exit and entry for each intervention group, respectively. We used the following model:

*Rate_t_* = *β_0_* *+* *β_1_time_t_* *+* *β_2_Phase_t_* *+* *β_3_Post_t_* *+* *e_t_*

in which *time* represents the study week to capture the overall trend in HH rates. *Phase* was coded as a 0 to 1 indicator of preintervention and postintervention periods, respectively, to capture the interaction between the sign intervention and time. Finally, to capture the change in slope, the *Post* variable was coded 0 in the baseline period and then sequentially numbered for the weeks after sign intervention.

Model diagnostics, including residual and autocorrelation plots, were used to evaluate the appropriateness of the model. Intercept changes and slope trends were reported as relative effects compared with the point estimates at the beginning of each phase. The overall intervention effect was estimated as the relative value of the point estimates with the coefficients of intercept and postintervention slope changes compared with point estimates without these coefficients. Bootstrapping methods were used to estimate the 95% CIs of relative changes.^27^

All analyses were performed using SAS Enterprise Guide statistical software version 7.1 (SAS Institute). *P* values were 2-tailed, and statistical significance was set at less than .05.

## Results

Table 1 presents HH activity by various observation characteristics including site, unit type, isolation status, season, and clinical staff type. During the baseline period, a total 4770 HH events of 9755 HH opportunities (48.9%) were observed at room entry, and 6439 HH events of 10 095 HH opportunities (63.8%) were observed at room exit. After randomization, 3057 HH events of 6100 HH opportunities (50.1%) at room entry and 4087 events of 6265 HH opportunities (65.2%) at room exit were observed during the intervention period.

**Table 1.** Hand Hygiene Adherence Stratified by Observation Context

Variable	HH Events, No. (%)							
	Room Entry				Room Exit			
	Baseline	No Change Group	Monthly Change Group	Weekly Change Group	Baseline	No Change Group	Monthly Change Group	Weekly Change Group
HH opportunies, No.	9755	2305	1781	2014	10 095	2412	1791	2062
Patient under isolation								
No	3904 (49.1)	1028 (52.7)	754 (50.5)	825 (50.8)	5056 (61.4)	1287 (63.8)	963 (64.0)	1081 (64.4)
Yes	866 (48.0)	164 (46.5)	125 (43.3)	161 (41.4)	1383 (74.1)	285 (72.3)	210 (73.4)	261 (68.2)
Site^a^								
1	754 (55.8)	331 (55.6)	NA	146 (54.7)	927 (63.6)	446 (68.0)	NA	167 (62.8)
2	63 (13.6)	8 (6.2)	6 (6.0)	11 (10.4)	347 (68.3)	138 (76.2)	89 (68.5)	106 (75.2)
3	383 (44.3)	37 (33.0)	209 (44.1)	125 (40.9)	573 (67.3)	58 (50.9)	291 (64.5)	178 (60.1)
4	563 (59.2)	242 (65.4)	NA	123 (55.9)	674 (71.2)	252 (73.0)	NA	159 (69.7)
5	321 (23.2)	44 (14.3)	58 (23.7)	36 (14.3)	662 (45.8)	120 (36.9)	115 (45.1)	84 (36.8)
6	876 (58.3)	89 (70.1)	173 (71.5)	124 (60.5)	1112 (69.4)	126 (83.4)	208 (79.7)	173 (75.9)
7	304 (43.7)	20 (39.2)	47 (49.0)	73 (49.7)	416 (57.1)	31 (57.4)	56 (60.9)	78 (52.4)
8	833 (73.9)	163 (71.8)	199 (74.5)	275 (72.0)	854 (77.7)	154 (69.1)	178 (75.1)	312 (77.2)
9	673 (47.6)	258 (66.8)	187 (52.4)	73 (56.2)	874 (60.1)	247 (68.0)	236 (64.7)	85 (69.7)
Unit type								
Intensive care unit	1032 (44.6)	238 (45.3)	171 (43.4)	150 (35.3)	1544 (62.7)	368 (64.7)	266 (64.6)	275 (58.6)
Medical	1321 (45.8)	437 (45.8)	262 (59.3)	161 (42.8)	1835 (63.1)	581 (59.6)	331 (73.6)	249 (66.8)
Surgical	698 (50.1)	145 (67.4)	71 (33.8)	144 (56.3)	876 (61.3)	161 (75.6)	128 (56.1)	170 (62.0)
Medical and surgical	679 (55.1)	104 (60.8)	126 (34.6)	366 (63.9)	825 (64.7)	120 (65.6)	205 (57.8)	406 (70.9)
Other	1040 (53.9)	268 (61.1)	249 (67.1)	165 (43.0)	1359 (67.3)	342 (72.5)	243 (70.2)	242 (64.9)
Season								
Fall	1038 (46.2)	589 (52.2)	432 (47.1)	440 (45.8)	1445 (61.8)	753 (64.8)	569 (62.9)	595 (61.9)
Winter	1846 (49.9)	22 (29.7)	62 (37.4)	43 (41.4)	2469 (64.7)	39 (48.2)	105 (62.5)	80 (64.0)
Spring^b^	1611 (50.9)	NA	NA	NA	2090 (63.8)	NA	NA	NA
Summer	275 (43.2)	581 (52.7)	385 (55.2)	503 (53.0)	435 (65.4)	780 (66.7)	499 (69.5)	667 (68.4)
Worker type								
Physician^c^	677 (49.1)	200 (53.6)	121 (45.2)	132 (53.2)	832 (60.0)	216 (58.5)	149 (59.1)	163 (63.7)
Nurse^d^	3438 (49.5)	837 (52.4)	662 (51.3)	703 (47.5)	4762 (65.4)	1147 (67.2)	892 (67.8)	1019 (65.9)
Other clinical staff^e^	655 (45.9)	155 (46.6)	96 (43.2)	151 (52.8)	845 (59.4)	209 (62.2)	132 (59.2)	160 (61.5)

Abbreviations: HH, hand hygiene; NA, not available.

^a^ For anonymity, sites are identified with numbers.

^b^ Because the intervention period lasted from June through December, no observations were obtained during the spring for this phase of the study.

^c^ Includes physicians and medical students.

^d^ Includes nurses (registered, licensed practitioners, and those with a bachelor of science degree in nursing), nursing students, patient care technicians, and nursing assistants.

^e^ Includes dieticians, nutritionists, infusion team, nurse practitioners, physician assistants, pharmacists, pharmacy students, radiology technicians, rehabilitation services, respiratory therapists, social workers, and unknown clinical staff.

### Change in HH Adherence During the Intervention Period

**Figure 2.**
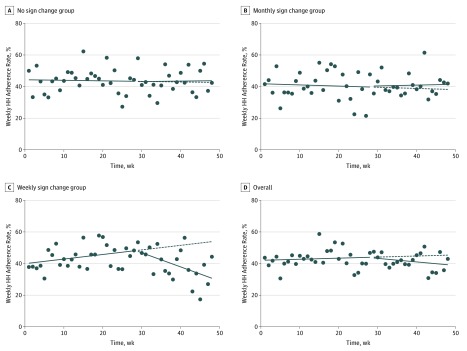
Hand Hygiene (HH) Adherence Rates at Room Entry Solid lines prior to the break indicate observed HH adherence rates prior to the intervention; solid lines after the break, observed HH adherence rates based on the intervention; dashed lines, predicted HH adherence rates if the intervention was not implemented; and dots, aggregate HH adherence rates per week.

**Figure 3.**
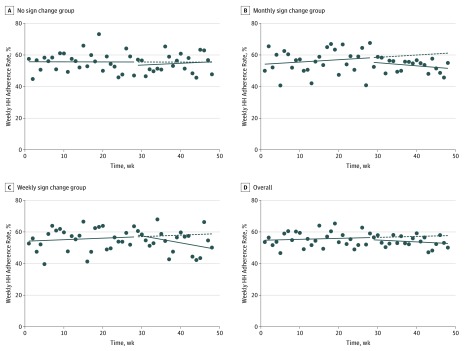
Hand Hygiene (HH) Adherence Rates at Room Exit Solid lines prior to the break indicate observed HH adherence rates prior to the intervention; solid lines after the break, observed HH adherence rates based on the intervention; dashed lines, predicted HH adherence rates if the intervention was not implemented; and dots, aggregate HH adherence rates per week.

Changes in HH adherence at room entry and exit by intervention group and overall are presented in Table 2. Minimal overall differences in HH adherence were observed between baseline and after intervention implementation when investigated as either a change in intercept (immediate change) or as a change in slope. When examined separately by group, no significant changes were found for units that did not change signs or for those that changed signs monthly. However, for units assigned to change signs on a weekly basis, there was a significant reduction in HH adherence during the intervention period. Specifically, the slope for HH adherence declined for room entry (−1.9% [95% CI, −2.7% to −0.8%] per week) and exit (−0.8% [95% CI, −1.5% to 0.1%] per week), reflecting a worsening in HH adherence over time (Figure 2 and Figure 3).

**Table 2.** Effect of the Intervention on Changes in Hand Hygiene Adherence Rates

Type of Adherence	Immediate Change, **%** (95% CI)	*P* Value for Immediate Change	Estimated Slope/wk, % (95% CI)		*P* Value for Slope Change	Estimated Relative Effect at the End of Study, % (95% CI)
			Preintervention	Postintervention		
Entry	−1.2 (−15.7 to 14.9)	.87	0.2 (−0.4 to 0.9)	−0.5 (−1.4 to 5.7)	.26	−19.9 (−44.5 to 12.6)
Exit	−2.6 (−10.6 to 5.8)	.44	0.1 (−0.2 to 0.5)	−0.2 (−0.7 to 0.4)	.29	−11.6 (−26.3 to 5.5)
**Subgroup Analysis**						
No sign change						
Entry	−0.4 (−20.5 to 23.0)	.97	−0.1 (−0.8 to 0.8)	0.1 (−1.2 to 1.8)	.86	4.0 (−34.5 to 66.1)
Exit	−3.7 (−16.4 to 10.2)	.58	0 (−0.5 to 0.5)	0.2 (−0.7 to 1.3)	.68	2.6 (−22.3 to 35.6)
Monthly sign change						
Entry	1.8 (−21.5 to 29.7)	.88	−0.2 (−1.0 to 0.8)	0.1 (−1.3 to 2.1)	.74	11.5 (−34.3 to 94.0)
Exit	−5.2 (−16.2 to 6.7)	.23	0.3 (−0.2 to 0.8)	−0.4 (−1.1 to 0.5)	.06	−21.4 (−40.3 to 1.7)
Weekly sign change						
Entry	−1.3 (−17.9 to 17.4)	.84	0.7 (−0.1 to 1.8)	−1.9 (−2.7 to −0.8)	<.001	−64.9 (−86.5 to −38.0)
Exit	1.8 (−11.1 to 16.1)	.73	0.2 (−0.3 to 0.8)	−0.8 (−1.5 to 0.1)	.02	−24.9 (−46.3 to 2.3)

## Discussion

This study was designed to examine whether the frequency of changing reminder signs affected inpatient health care workers’ HH adherence. We hypothesized that changing signs on a weekly or monthly basis would increase the noticeability of the signs and reduce the potential for habituation. However, the frequency of changing signs had minimal effect on HH adherence overall. The most frequent sign changing schedule (ie, weekly) was associated with a decrease in HH adherence of 1.9% per week at room entry and 0.8% per week at room exit. Although counterintuitive, it may be that changing signs more frequently reduced rather than increased their salience compared with less frequent changes. It is also possible that the observed patterns simply reflected chance findings rather than a true association.

Considering the positive effects of reminder signs on HH adherence observed in some prior studies,^28,29^ the reasons for the lack of a positive effect for changing communications and health behavior theory–informed point-of-care signs on HH behavior are not clear. Given the abundance of signs on hospital units, the frequency of changing signs may be too weak of a signal to have a clinically meaningful effect. It also may simply be that passive reminders, such as signs, even if well designed and based on theory-grounded and evidence-based constructs, are insufficient by themselves to significantly affect HH in this context.^9^ This is in agreement with findings that have suggested that multicomponent intervention bundles combining several different strategies are associated with the most meaningful and lasting improvements in HH adherence^30,31^ and raise questions regarding the role of reminder signs in multicomponent bundles. In an era of constrained resources, understanding what activities do not lead to desired improvements in health care delivery is arguably as important as understanding what does work. In this study, we evaluated a common practice that is time-consuming (manual sign exchange at every ABHS dispenser) and sometimes logistically challenging (eg, creating signs holders that easily allow interchange). Our data suggest that resources could be allocated elsewhere without a decrement in HH adherence rates.

Additionally, given the inherent complexity involved in implementing interventions in clinical practice, organizational support and commitment by local leadership are likely needed to promote successful behavioral change targeting HH.^32^ A 2002 study^33^ of an intervention involving visual performance feedback similarly reported no impact on HH in the absence of hospital leadership and organizational commitment. Therefore, the absence of more structured, dedicated efforts to engage hospital administrators and other key leaders may have contributed to the lack of intervention effects.

Visual cues need to be more conspicuous than the surrounding environment to attract attention and elicit the desired behavior.^18,34^ In the busy hospital environment, that can be difficult to achieve in the context of important patient care responsibilities and environmental stimuli that compete for clinical staff’s attention and tax their attentional capacity.^35^ Therefore, characteristics that increase the conspicuity of the cues may improve their ability to generate attention. Cues with dynamic features, such as flashing lights, alarm sounds, and sensors to activate them, appear more effective at changing behavior than static signs.^36^ Studies by D’Egidio et al^35^ and Rashidi et al^37^ found that adding flashing lights to ABHS dispensers located at the entrance to a hospital was associated with increased HH behavior. However, it is unclear whether reminders with these design characteristics are feasible for use in an inpatient clinical environment.

Owing to hospital regulations, signs and ABHS dispensers included in this study were located at a distance from patient care activities (room entry), which also may have decreased their effect and use.^38^ Positioning ABHS dispensers and reminder signs in locations that are more proximal to patient care may be more successful. The relatively small size of the signs, which measured 21.59 cm by 27.94 cm at 2 hospitals, 13.97 cm by 10.79 cm at 6 hospitals, and 7.62 cm by 10.16 cm at 1 hospital, may also have reduced their effectiveness. Finally, the fact that the location of the signs did not vary but instead was kept consistent may have reduced their ability to attract attention.

### Strength and Limitations

Strengths of this study include the cluster randomized design, the number of geographically dispersed hospitals, inclusion of several types of inpatient units, use of theory-guided messages for promoting HH, and covert observation of health care worker HH behavior. Focusing on a single intervention approach (ie, changing signs) also allowed us to isolate and evaluate the effectiveness of a strategy commonly included as part of multicomponent HH intervention bundles, something that has not been possible in most prior studies to our knowledge and which may have hampered efforts to identify the most effective combination of strategies.^8,30,39^

Our study also has limitations. The first was that, owing to stipulations of the federal labor unions that represented most of the health care workers included in this study, we were not able to document HH adherence at the level of the individual health care worker or to track them from one room to another. A health care worker’s HH behavior is likely to be consistent from one patient to another, so observations involving the same health care worker would likely be correlated. Observations involving different health care workers attending the same patient would also likely be correlated. However, because the identity of the health care worker could not be documented as part of the monitoring process, observations were assumed to be independent for purposes of analysis. Ignoring these sources of correlation may have resulted in an underestimate of SEs and inflated the required sample size. However, we did conduct a separate set of analyses based on general estimating equations at the level of the individual patient encounter. These findings did not differ meaningfully from those reported here, so results were not presented here. Second, because it was determined that the study would not be able to attain the target number of observations within a reasonable time while still addressing the aims of the larger cluster randomized trial, the intervention period was not extended beyond the originally planned 6-month interval. As a result, the study was underpowered to detect a significant treatment effect. However, the minimal differences in HH adherence that were observed between groups suggest that insufficient power was not likely to be a significant contributor to the null findings. Third, although the study included a relatively large number of observations, we were able to sample only a small proportion of all HH opportunities that took place on each unit. Furthermore, observation activity was largely restricted to weekdays and the day shift. Fourth, although observations were designed to be covert, there was still potential for behavioral reactivity to being observed if health care workers noticed they were being watched. Fifth, all sites were US Department of Veterans Affairs medical centers; thus, generalizability to other settings is uncertain.

## Conclusions

This randomized cluster trial found that use of theory-informed signs was not associated with changes in HH adherence among inpatient health care workers. The frequency with which the signs were changed also had minimal impact on HH rates overall, with statistically significant effects limited to units assigned to change signs weekly, and, contrary to expectations, frequent (ie, weekly) sign changes were associated with a statistically significant worsening rather than improvement in HH adherence. Our findings suggest that changing reminder signs on a regular basis was not an adequate cue for HH behavior and, under some circumstances, may adversely affect adherence, bringing into question the use of reminder signs to improve HH rates. Future efforts to improve health care worker HH adherence using visual prompts or cues should consider incorporating design elements to better attract and sustain attention. Alternative health communication and messaging strategies should also be evaluated.

## Notes

Supplement 1. **eAppendix.** Examples of Hand Hygiene Reminder Signs **eTable.** Observational Activity by Study Site, Unit, and Other Characteristics

Supplement 2. **Trial Protocol**

Supplement 3. **Data Sharing Statement**
