# Supplementary material for: Effect of Frequency of Changing Point-of-Use Reminder Signs on Health Care Worker Hand Hygiene Adherence: A Cluster Randomized Clinical Trial
Source: JAMA Netw Open. 2019 Oct 23;2(10):e1913823. doi: 10.1001/jamanetworkopen.2019.13823 (PMC6820039; doi:10.1001/jamanetworkopen.2019.13823)
Supplement: Supplement 1. — eAppendix. Examples of Hand Hygiene Reminder Signs eTable. Observational Activity by Study Site, Unit, and Other Characteristics [file jamanetwopen-2-e1913823-s001.pdf]

Supplementary Online Content

Vander Weg MW, Perencevich EN, O'Shea AMJ, et al. Effect of frequency of changing point-of-use reminder signs on health care worker hand hygiene adherence: a cluster randomized clinical trial. *JAMA Netw Open*. 2019;2(10):e1913823. doi:10.1001/jamanetworkopen.2019.13823

eAppendix. Examples of Hand Hygiene Reminder Signs

eTable. Observational Activity by Study Site, Unit, and Other Characteristics

This supplementary material has been provided by the authors to give readers additional information about their work.

© 2019 Vander Weg MW et al. *JAMA Network Open.*

eAppendix. Examples of Hand Hygiene Reminder Signs

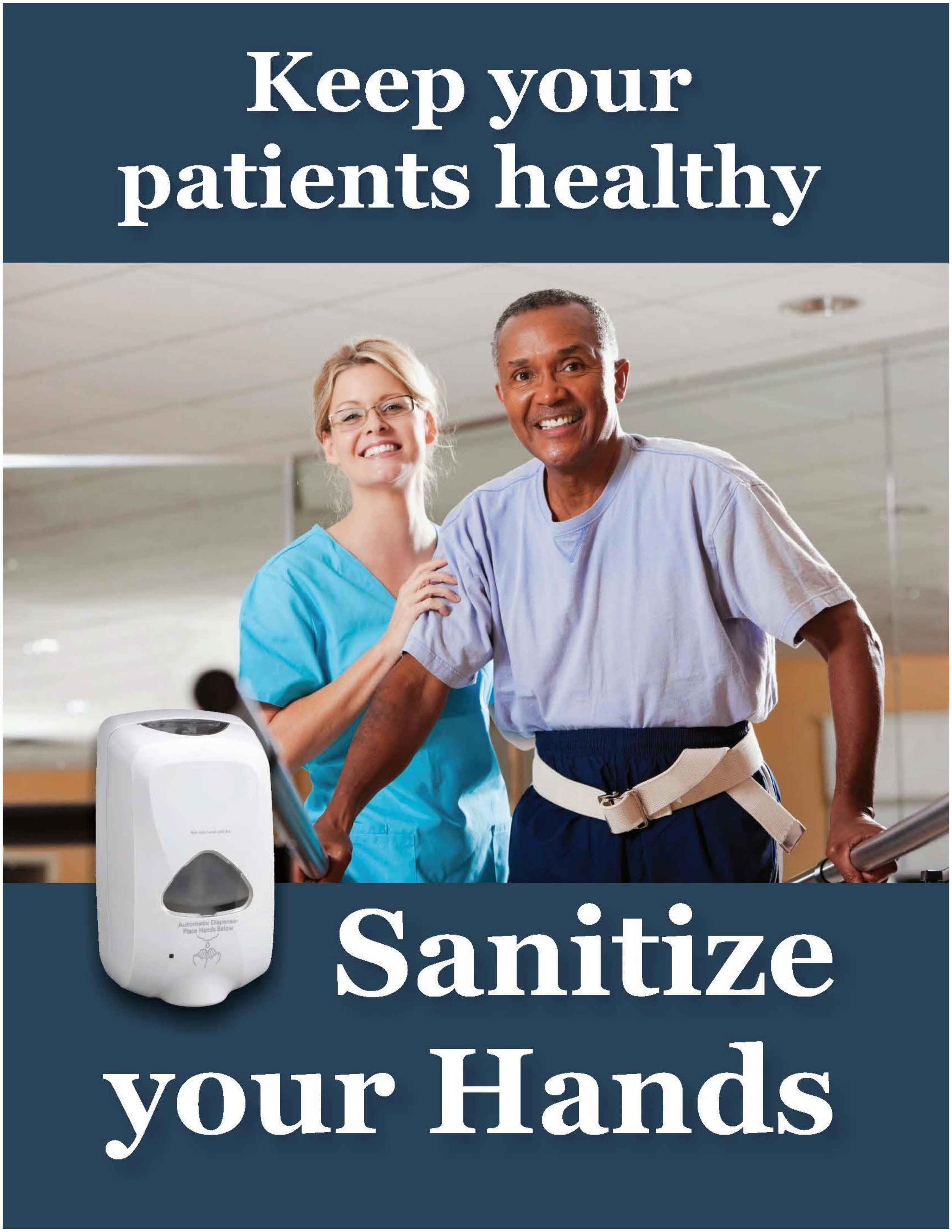

© 2019 Vander Weg MW et al. *JAMA Network Open.*

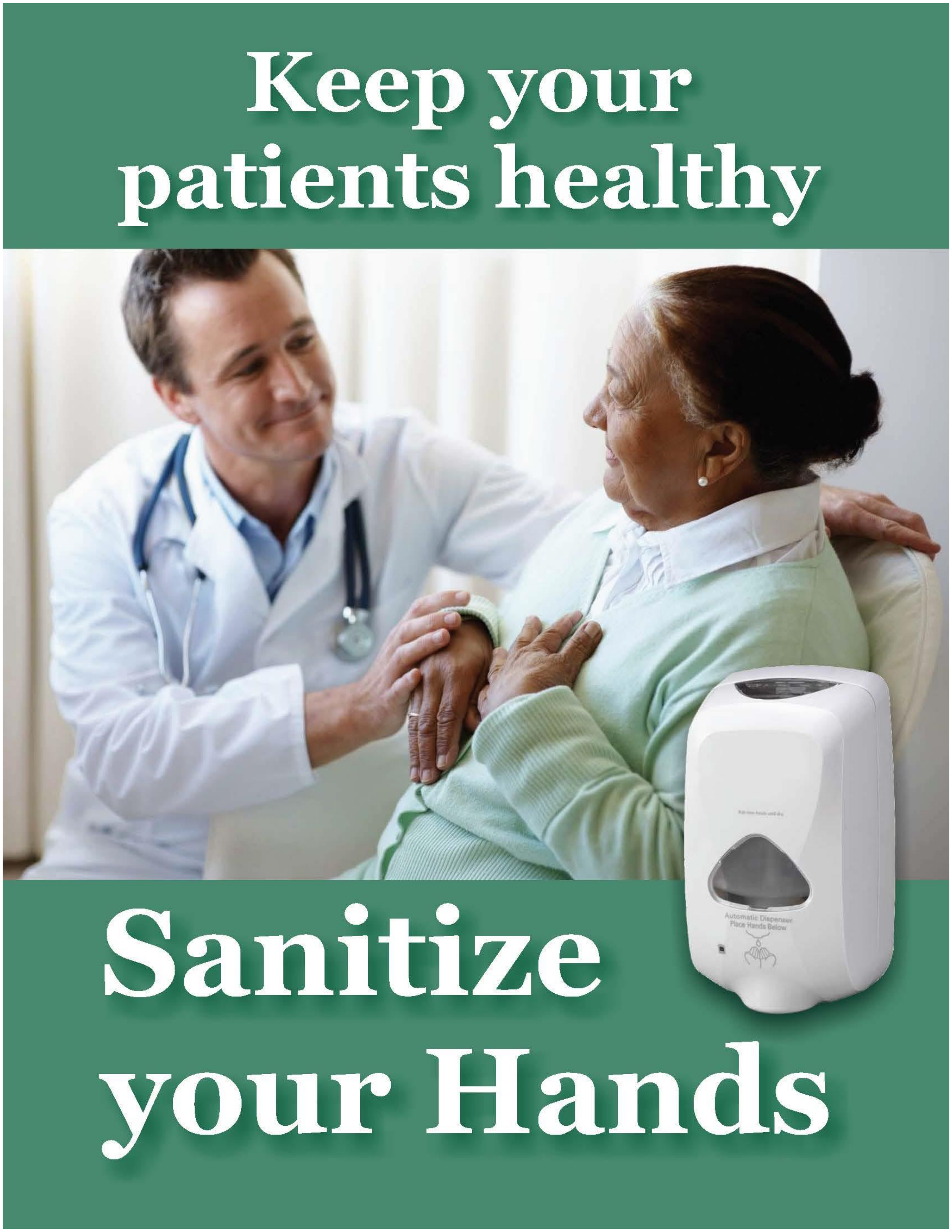

© 2019 Vander Weg MW et al. *JAMA Network Open.*

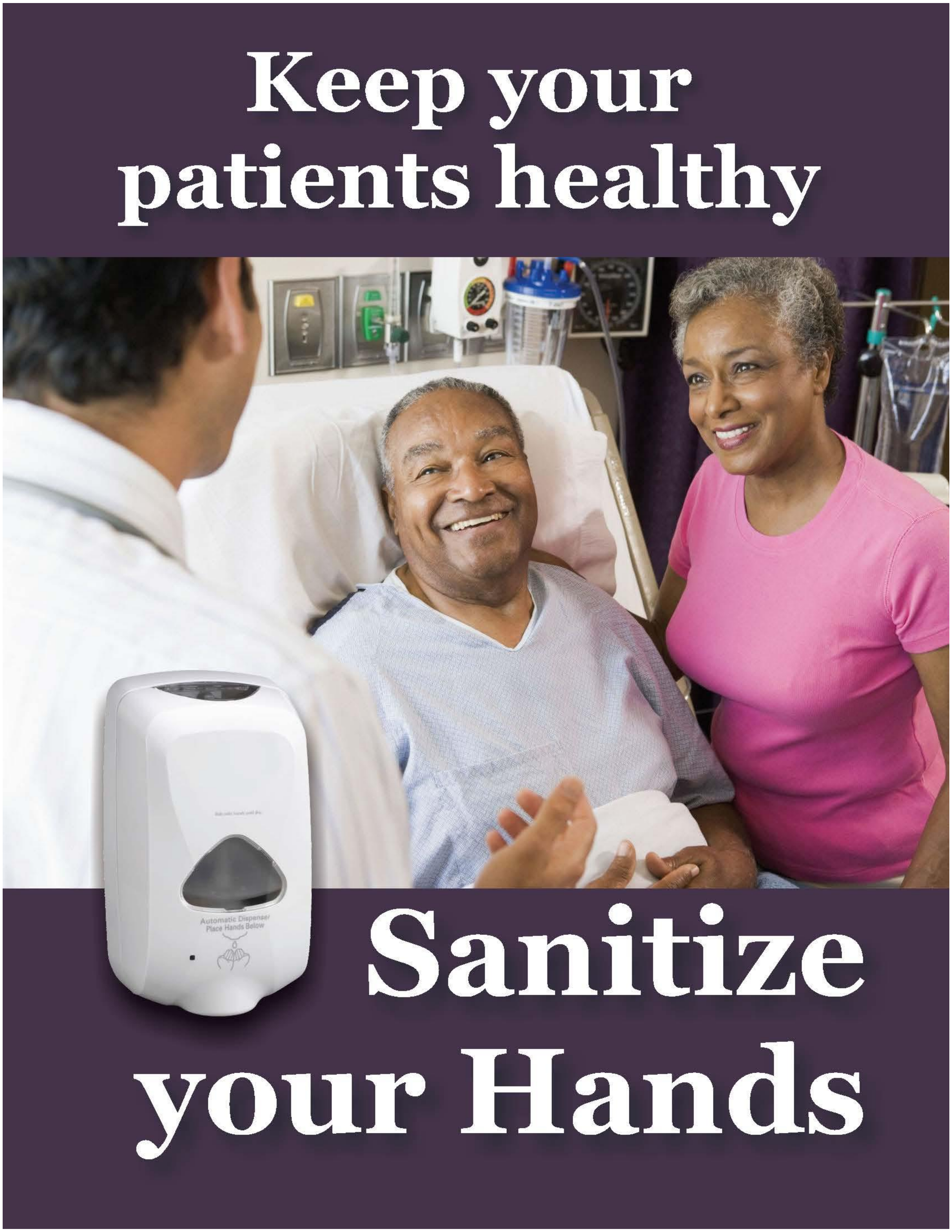

© 2019 Vander Weg MW et al. *JAMA Network Open.*

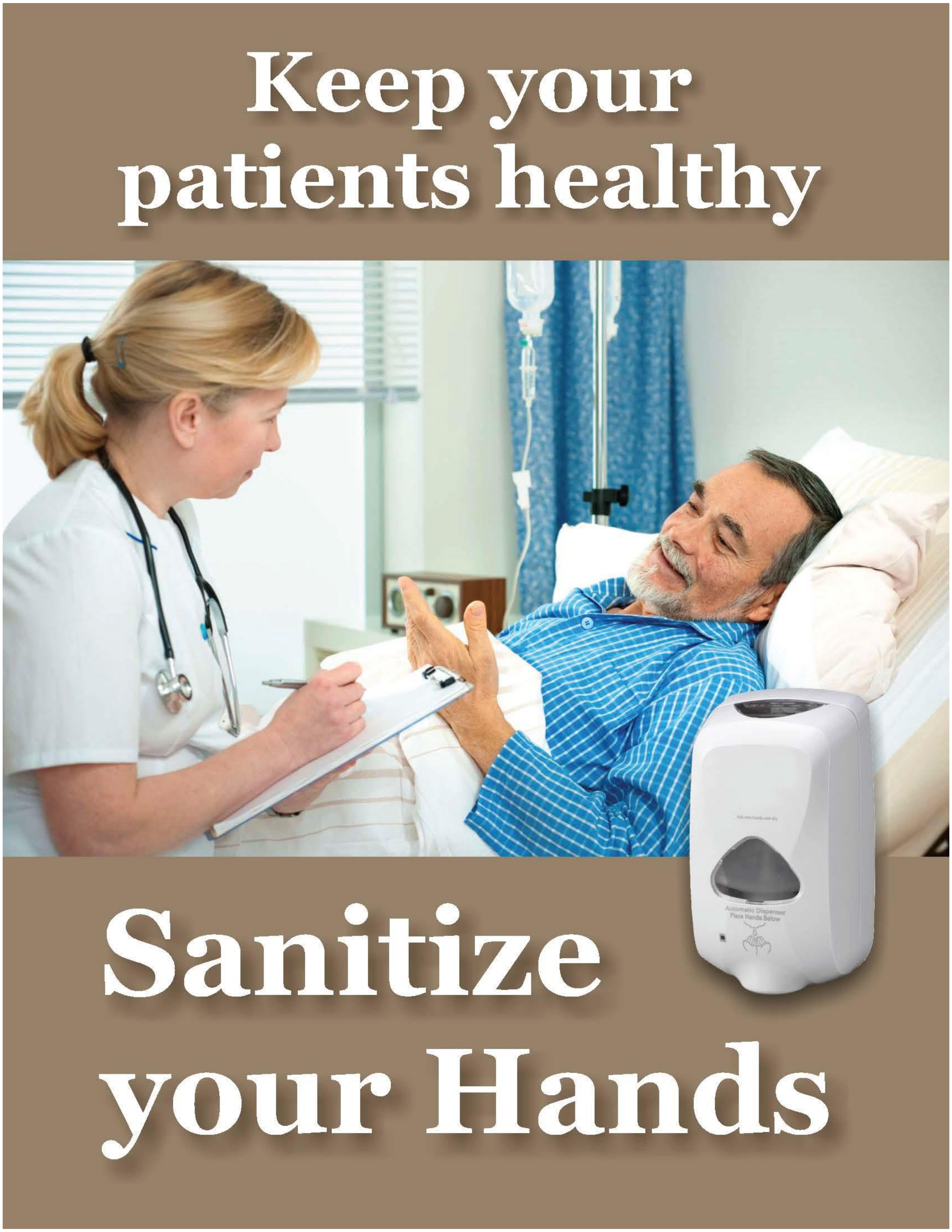

© 2019 Vander Weg MW et al. *JAMA Network Open.*

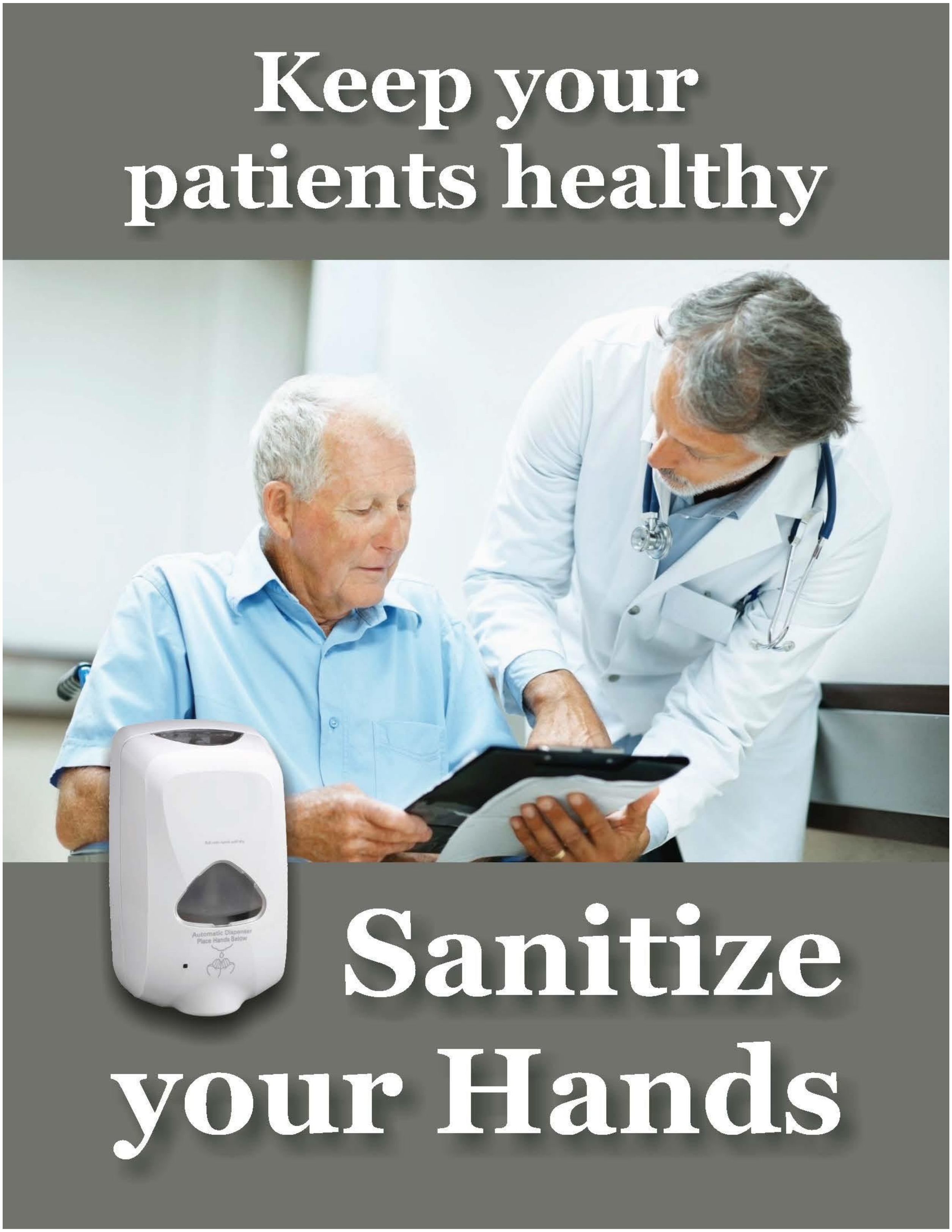

© 2019 Vander Weg MW et al. *JAMA Network Open.*

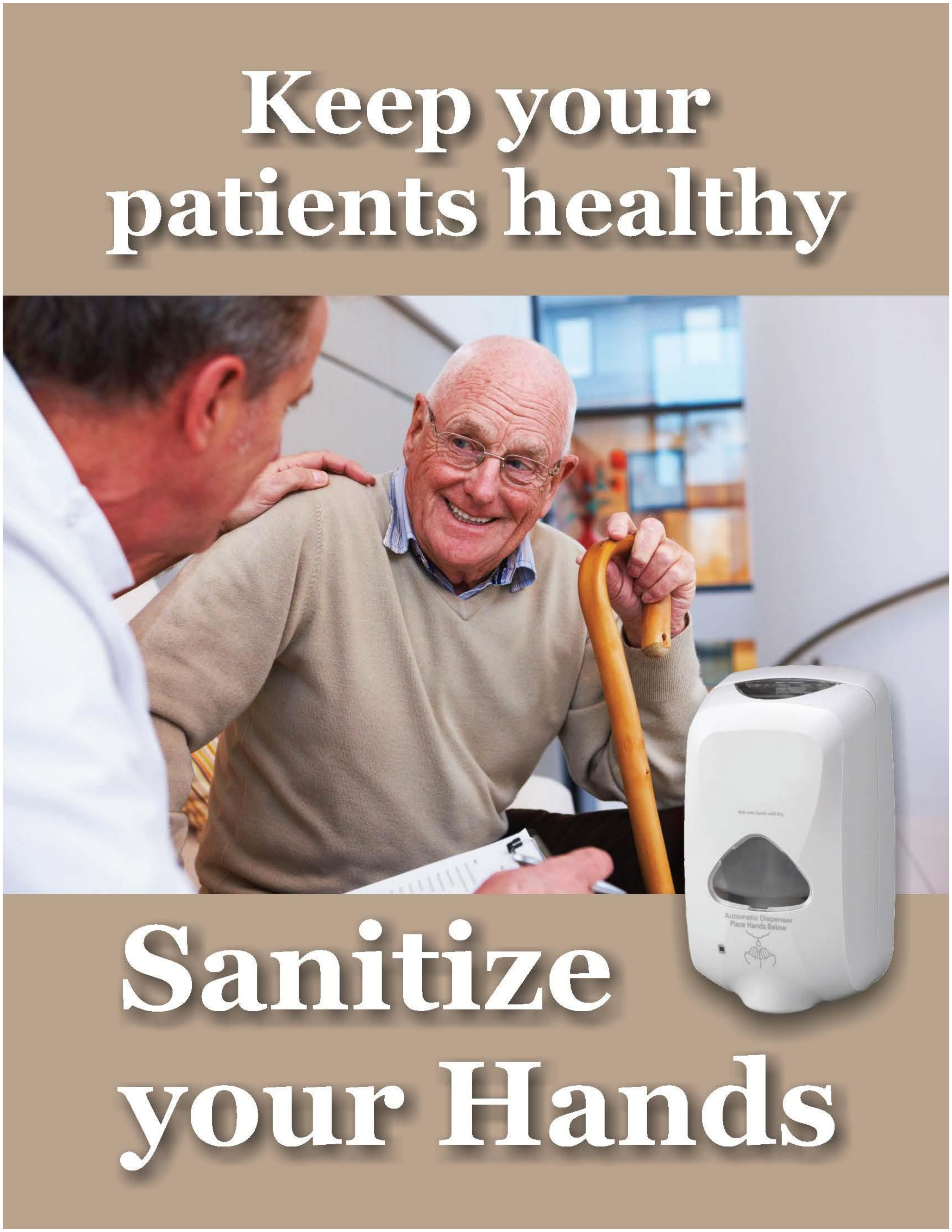

© 2019 Vander Weg MW et al. *JAMA Network Open.*

eTable. Observational Activity by Study Site, Unit, and Other Characteristics

**Variable**		**N (%)**	**Study Phase (N [%])**	
		**Overall (N = 18904)**	**Baseline (N = 11666)**	**Intervention (N = 7238)**
Site	Site 1	2267 (12.0)	1358 (11.6)	909 (12.6)
	Site 2	2140 (11.3)	1286 (11.0)	854 (11.8)
	Site 3	1961 (10.4)	1219 (10.5)	742 (10.3)
	Site 4	2219 (11.7)	1295 (11.1)	924 (12.8)
	Site 5	2204 (11.7)	1363 (11.7)	841 (11.6)
	Site 6	1662 (8.8)	1146 (9.8)	516 (7.1)
	Site 7	1806 (9.6)	1272 (10.9)	534 (7.4)
	Site 8	2321 (12.3)	1368 (11.7)	953 (13.2)
	Site 9	2324 (12.3)	1359 (11.7)	965 (13.3)
Unit Type	ICU	3931 (20.8)	2486 (21.3)	1445 (20.0)
	Medical	6091 (32.2)	3777 (32.4)	2314 (32.2)
	Surgical	2877 (15.2)	1819 (15.6)	1058 (14.6)
	Medical / Surgical	2469 (13.1)	1390 (11.9)	1079 (14.9)
	Other	3536 (18.7)	2194 (18.8)	1342 (18.5)
Isolation Type	Contact	3352 (17.7)	2029 (17.4)	1323 (18.3)
	Droplet	183 (1.0)	144 (1.2)	39 (0.5)
	Airborne	77 (0.4)	40 (0.3)	37 (0.5)
	Enteric	369 (2.0)	236 (2.0)	133 (1.8)
	Other	316 (1.7)	194 (1.7)	122 (1.7)
Isolation (Yes)		4249 (22.5)	2608 (22.4)	1641 (22.7)
No Activity (Yes)		6723 (35.6)	4228 (36.2)	2495 (34.5)
Intervention Group[2]	No Sign Change	7213 (38.2)	4388 (37.6)	2825 (39.0)
	Weekly Sign Change	6238 (33.0)	3894 (33.4)	2344 (32.4)

© 2019 Vander Weg MW et al. *JAMA Network Open.*

	Monthly Sign Change	5453 (28.9)	3384 (29.0)	2069 (28.6)
Season	Fall	6009 (31.8)	2422 (20.8)	3587 (49.6)
	Winter	4791 (25.3)	4368 (37.4)	423 (5.8)
	Spring	3955 (20.9)	3955 (33.9)	0 (0.0)
	Summer	4149 (22.0)	921 (7.9)	3228 (44.6)

[1]Reflects observation activity for clinical staff or where no activity was reported.
[2]Isolation types are not mutually exclusive.

© 2019 Vander Weg MW et al. *JAMA Network Open.*
