# Supplementary material for: Effect of Frequency of Changing Point-of-Use Reminder Signs on Health Care Worker Hand Hygiene Adherence: A Cluster Randomized Clinical Trial
Source: JAMA Netw Open. 2019 Oct 23;2(10):e1913823. doi: 10.1001/jamanetworkopen.2019.13823 (PMC6820039; doi:10.1001/jamanetworkopen.2019.13823)
Supplement: Supplement 2. — Trial Protocol [file jamanetwopen-2-e1913823-s002.pdf]

Building Optimal Hand Hygiene Bundle: A Mixed Methods Approach

Funding Agency: Department of Veterans Affairs

Principal Investigator/Study Chair: Heather S. Reisinger, PhD

Version 1 7/15/2013

Abstract

"Hand hygiene is the single most important measure to prevent transmission of infectious organisms" (*VHA Directive 2010-006, MRSA Prevention Initiative*). Despite its fundamental place in infection prevention, compliance rates with hand-hygiene protocols remain substantially below target levels. This project will advance the science of hand-hygiene compliance interventions by developing an evidence-based bundle of effective strategies. While many have advocated for a multifaceted bundle as the most effective approach to improving hand-hygiene compliance, no studies have systematically identified an optimal combination of interventions. As such, infection prevention programs continue to use a variety of hand-hygiene interventions without clear evidence of which components in isolation or combination are most effective. The two specific aims and associated hypotheses of CREATE Project 2 include:

1) Identify combinations of hand-hygiene intervention strategies that optimize hand-hygiene compliance and that could form an evidence-based hand-hygiene bundle for VHA implementation.
   Hypothesis 1: Combinations of interventions will increase compliance rates more than single interventions.

Aim 1 will entail a 30-month cluster-randomized controlled trial that will sequentially test three individual hand-hygiene interventions – hand-hygiene point-of-use reminder signs to serve as an environmental cue to action, individual hand sanitizers, and health care worker hand cultures – to identify an optimal combination of interventions to increase hand-hygiene compliance. The trial will be conducted in 59 hospital units in 10 VA hospitals in order to test the efficacy of individual and then sequentially added interventions to determine their incremental impact on hand-hygiene compliance.

2) Identify institutional, organizational, ward/ICU, and individual level facilitators and barriers to implementing hand-hygiene interventions.
   Hypothesis 2: Facilitators and barriers will pattern around contextual factors such as level of leadership support and organization of infection control programs.

Aim 2 will entail a qualitative process evaluation that includes site visits to purposefully selected sites, semi-structured interviews, and observations to examine barriers and facilitators to the interventions and develop contextual insight for implementing and scaling-up the intervention at additional sites as a national initiative.

List of Abbreviations
MRSA: Methicillin-resistant *Staphylococcus aureus*
HAI: Hospital-associated infections
TJC: The Joint Commission
CDC: Centers for Disease Control
WHO: World Health Organization
VAMC: VA Medical Centers
VHA: Veterans Health Administration
HBM: Health Belief Model
CRT: Cluster Randomized Trials
RCT: Randomized Control Trials
CLCs: Community Living Centers
CDW: Corporate Data Warehouse
CPRS: Computerized Patient Record System
IPEC: VA Inpatient Evaluation Center
VINCI: Veterans' Informatics & Computing Infrastructure
APACHE: Acute Physiology, Age, Chronic Health Evaluation
SQL: Structured Query Language
NIDS: National Infectious Disease Program Office
MDRO: MRSA / Multidrug-resistant Program Office
COHIC: National Center for Occupational Health and Infection Control
NHSN: National Healthcare Safety Network
IDPIO: Infection: Don't Pass It On
OIG: Office of Inspector General
HCWs: Health Care Workers
IC: Infection Control
HH: Hand Hygiene
MeSH: Medical Subject Headings
ICPs: Infection Control Professionals

Contents

Protocol Title: 5
1.0 Study Personnel 5
2.0 Introduction 6
3.0 Objectives 17
4.0 Resources and Personnel 18
5.0 Study Procedures 19
5.1 Study Design 19
5.2 Recruitment Methods 26
5.3 Informed Consent Procedures 27
5.4 Inclusion/Exclusion Criteria 28
5.5 Study Evaluations 29
5.6 Data Analysis 29
5.7 Withdrawal of Subjects 33
6.0 Reporting 34
7.0 Privacy and Confidentiality 35
8.0 Communication Plan 36
9.0 References 37

Protocol Title: Building Optimal Hand Hygiene Bundle: A Mixed Methods Approach

1.0 Study Personnel

Principal Investigator/Study Chair:
Heather Reisinger, PhD, Iowa City VA Medical Center heather.reisinger@va.gov

Co-Investigators:
Eli Perencevich, MS, MD, Iowa City VA Health Care System, eli.perencevich@va.gov
Mark Vander Weg, PhD, Iowa City VA Health Care System, mark.vanderweg@va.gov

Co-Investigators/Local Site Investigators:
Gio Barraco, MD, Miami VA Health Care System, gio.baracco@va.gov
Marvin Bittner, MD, VA Nebraska-Western Iowa Health Care System, marvin.bittner@va.gov
Sue Bradley, MD, VA Ann Arbor Health Care System, sbradley@umich.edu
Graeme Forrest, MD, Portland VA Medical Center, Graeme.forrest2@va.gov
Kalpana Gupta, MD, Boston VA Health Care System, Kalpana.gupta@va.gov
Jose Cadena-Zuluaga, MD, South Texas Veterans Health Care System, jose.cadena-zuluaga@va.gov
Daniel Morgan, MD, VA Maryland Health Care System, Daniel.morgan2@va.gov
Michael Rubin, MD, VA Salt Lake City Health Care System, Michael.rubin2@va.gov
Joseph Thurn, MD, Minneapolis VA Health Care System, joseph.thurn@va.gov

Collaborators:
Judy Streit, Iowa City VA Health Care System
Sarah Klein, VA Ann Arbor Health Care System
Luci Leykum, South Texas Veterans Health Care System

2.0 Introduction

A. Background

Hand hygiene is key to preventing and controlling methicillin-resistant *Staphylococcus aureus* (MRSA) and other hospital-acquired infections (HAIs). This project will advance the science of hand hygiene compliance interventions by developing an evidence-based bundle of effective strategies. While many have advocated for a multifaceted bundle as the most effective approach to improving hand hygiene compliance, no studies have systematically identified an optimal combination of interventions.[2, 7-9] As such, infection prevention programs continue to use a variety of hand hygiene interventions without clear evidence of which components in isolation or combination are most effective.

<u>Hand Hygiene, Infection Control, and Compliance Rates</u>: Hand hygiene is widely considered the most effective method of preventing HAIs.[1-2] Multiple studies have demonstrated that increases in hand hygiene compliance lower the rates of infection in hospital settings.[10-13] Yet, hand hygiene compliance remains persistently low.[1-2, 14] In the latest systematic review of hand hygiene compliance, the average compliance rate was 40%.[3] In our own work, we have obtained similar findings. In one study of VA and non-VA hospitals, hand hygiene compliance averaged 32.9% for room entry and 50.8% for exit.[15] However, the average compliance rate reported by infection control programs at the same sites was 87.2%. Thus, rates obtained via rigorous observation methods following established guidelines were much lower than those found in routine practice, where observation methods: 1) are much more variable, 2) frequently do not follow recommended methods,[16-18] and 3) are subject to considerable inflation, likely due, at least in part, to the Hawthorne effect.[19-20] The Joint Commission's (TJC) revision of their National Patient Safety Goal for hand hygiene (NPSG.07.01.01) reflects a similar realization. As Mark Chassin, President of TJC stated, "In April 2009—at the beginning of the [hand hygiene] project—performance was collectively at 48%...It's interesting that a number of the hospitals were misled by faulty data to believe that they were doing as well as, say, 85%, at baseline rather than 48%."[21] The 2012 TJC National Patient Safety Goal for hand hygiene (NPSG.07.01.01) now states: "Comply with either the current Centers for Disease Control and Prevention (CDC) hand hygiene guidelines or the current World Health Organization (WHO) hand hygiene guidelines. Set goals for improving compliance with hand hygiene guidelines. Improve compliance with hand hygiene guidelines based on established goals." However, this same goal does not require facilities to follow evidence-based guidelines on measuring hand hygiene, despite TJC's publication of such guidelines.[18] In addition, little guidance exists in the TJC's goal on which intervention strategies improve hand hygiene compliance rates—and prevent infectious disease.

<u>Hand Hygiene Clinical Practice Guidelines and VHA Directives</u>: Currently, VHA Directive (2011-007) "Required Hand Hygiene Practices" provides regulations on hand hygiene practice in VHA. This Directive is primarily a reiteration of CDC and WHO guidelines for a VHA context, including when to use antimicrobial soap or alcohol-based hand rub, hand hygiene practices related to glove usage, availability and placement of hand hygiene products, and where to obtain VA *Infection: Don't Pass It On* campaign materials from the Office of Public Health

(http://www.publichealth.va.gov/infectiondontpassiton/). It also requires all VA facilities to have a written hand hygiene policy. What is missing in the Directive is guidance on the best interventions for improving hand hygiene. WHO guidelines provide some recommendations for interventions, but focus on multimodal, promotional campaigns at the hospital-system or country level, while also acknowledging science is lacking on which components of the campaign are essential for hand hygiene improvement.[2] VHA has made it a priority to develop a web-based Hand Hygiene Toolkit for Infection Control Professionals (ICPs) in the field,[22] "but there are few concrete practical strategies for complying with these guidelines."[23]

State of the Literature on Hand Hygiene Interventions: Systematic reviews confirm the lack of strong evidence regarding effective hand hygiene interventions.[8, 10, 24-26] In 2011, a Cochrane Review of hand hygiene interventions found only 4 studies that met their review criteria of randomized control trials, controlled clinical trials, controlled before and after studies, and interrupted time series analyses from 1980 to November 2009. The majority of the 133 potential studies that were identified were excluded due to their poor quality. Multiple systematic literature reviews have come to the same conclusion, citing factors that contribute to the poor quality of the studies such as small sample size, short follow-up duration, inconsistent outcomes measures, lack of or inappropriate control group, and lack of generalizability outside the particular ward or ICU.[8]

Another issue is that, although bundled, multimodal interventions are regarded as the state-of-the-art in hand hygiene improvement, many studies focused on single interventions.[27] Single intervention studies have tested education,[28-30] visual cues,[31-34] direct observation and feedback,[35-36] electronic and video monitoring and feedback,[20, 37-40] incentives and rewards,[41-42] and role modeling.[43-45] However, changing hand hygiene behavior is a complex endeavor. To this end, multimodal—or "bundled"—interventions that draw on behavioral change theory are considered the most effective.[46-48] Pittet and colleagues'[12] work in the University of Geneva hospital is often cited as an example of an effective multimodal hand hygiene intervention.[49-50] The intervention includes strategically-displayed, collaboratively-designed posters; performance feedback; distribution of individual hand sanitizer; alcohol hand-rub dispensers mounted to patient beds; and institutional support. In an evaluation of this intervention, overall compliance improved significantly over a 3-year period from 47.6% to 66.2% ($p<0.001$). With regard to HAI, infections decreased from 16.9% to 9.9% ($p=0.04$) and overall incidence of MRSA decreased from 2.16 to 0.93 episodes per 10,000 patient days ($p<0.001$) over a 4 year period.

The challenge is: What effect was associated with each individual intervention component, and did all components contribute meaningfully to enhanced hand hygiene rates? Further, when separate intervention components are combined, do they enhance the overall impact on hand hygiene compliance in an additive or synergistic manner, or do they detract from one another? The study only observed the combined effect of all components together. In addition, Pittet and colleagues'[12] work is frequently cited for its innovation, sustainability, and measured effect on HAIs, but it was a single-center study with potential lack of generalizability outside the particular Swiss hospital in which it was conducted. What is needed to advance the science of hand

hygiene are well-designed, multi-site studies that disentangle the components of bundled hand hygiene interventions.

Identifying Three Hand Hygiene Intervention Strategies: Based on our own systematic review, the VHA-wide hand hygiene survey, and our pilot data,(see Preliminary Studies Section) we have selected three interventions that are most likely to improve hand hygiene compliance. These interventions are 1) hand hygiene point-of-use reminder signs to serve as an environmental cue to action; 2) individual hand sanitizers, and 3) health care worker (HCW) hand cultures. Hand hygiene signs are the most commonly used intervention for their ease of use and low cost.[2, 24, 27] Further, our pilot data and two recent studies suggest theoretically-based signs have a positive effect on hand hygiene behavior.[33-34] However, these findings need to be validated in a larger, multi-site study. The second intervention strategy is the systematic disbursement of individual hand sanitizers to HCWs. Individual hand sanitizers are a component of many of the multimodal hand hygiene studies.[12, 51-52] Due to their frequent use in bundles, WHO guidelines recommend providing HCWs with individual hand sanitizer.[2] The VHA hand hygiene directive (2011-007) requires VA facilities to make individual hand sanitizers available to HCWs and according to our findings in the VHA-wide survey, over 90% of VA facilities comply. Yet, despite their wide-spread use, individual hand sanitizers have not been tested for their stand-alone or additive effect. The third strategy—the display of HCW hand cultures—is an innovative approach that has been piloted with success in quality improvement projects.[53-54] With this approach, HCWs are asked to place their hand on a blood agar culture plates, the organisms left by their hand are grown, a photograph is taken of the results, and then the photographs of the hands are displayed on a unit with unique identifiers. This allows HCWs to observe the organisms cultured from their own hands, while also viewing a collective picture of the healthcare team on that unit or ward. However, this approach has not yet been evaluated sufficiently in the literature—individually or combined as a bundle—to recommend wide adoption. In this proposal, we will test each of these strategies for their additive, synergistic, or even detracting effect to improve the evidence base for hand hygiene interventions.

Conceptual Model

Besides issues of design quality, much of the current hand hygiene literature calls for incorporating behavioral and organizational change theory in the development of hand hygiene interventions.[46, 55] Indeed, improving hand hygiene is fundamentally about changing behavior.[56] This proposal integrates theories from both areas to inform its intervention, including the Nested Theory of Structuration[57] and the Health Belief Model (HBM).[58] (See Figure 1.)

This proposal fits into and broadly draws from the overarching theoretical model of Nested Theory of Structuration in which individual action can build into patterns of interaction (teams) that are embedded in an organizational and institutional context.[57] In the larger CREATE proposal, each individual study reflects one of these levels to facilitate a comprehensive approach to the control and prevention of MRSA and other HAIs. Specifically, the hand hygiene study focuses on the *individual behavior* of HCWs disinfecting their hands, the surgical site infection study targets *patterns of interaction among the surgical team*, the Community Living Centers study examines *organizational context*, and finally the computer model integrates all of

the studies to understand the *institutional context* at a VISN-level. At the same time, each study must be cognizant of all four levels and how they mutually influence (and are influenced by) one another.

Figure 1. Framework for Understanding Individual HCW Behavior Situated in Organizational and Institutional Contexts

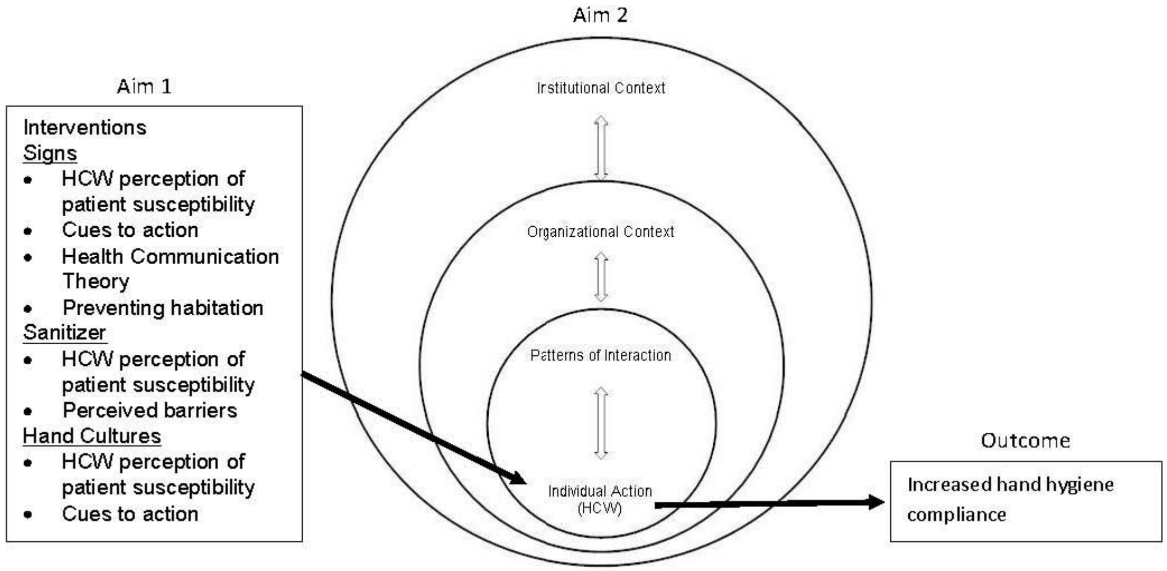

The Nested Theory of Structuration will guide our research questions, particularly in Aim 2, as we attempt to understand the impact of patterns of interactions among HCWs and the organizational (facility-level) and institutional (VA-level) context on individual hand hygiene behavior. For example, during observations of wards/units and infection control rounds, is there evidence of team interaction as a facilitator or barrier to hand hygiene compliance? In semi-structured interviews, does the infection control team articulate a sense of support from facility leadership? Is the infection control team aware of national hand hygiene policies? Are VA *Infection: Don't Pass It On* (IDPIO) posters prominently displayed on wards/units at the point of care? Answers to these questions will help address the broader question of: What is the "hand hygiene culture" of the facilities? In turn, this information will assist in tailoring the implementation plans for the interventions to address some of the known barriers and take advantage of existing facilitators.

Besides focusing on organizational and structural aspects that influence hand hygiene practices, we also draw on the HBM in the design of our intervention targeting individual HCW behavior and to guide our research questions in Aim 1. The HBM incorporates six constructs: perceived susceptibility, perceived severity, perceived benefits, perceived barriers, cues to action, and self-efficacy.[58] The traditional focus of the HBM has been to change patients' health behavior; however, the intended behavior change of this intervention involves HCWs. Work in this area suggests a need to shift perceived susceptibility from the person performing the behavior (HCW) to the individual most susceptible (patient). Therefore, the intervention concentrates on changing HCW behavior by focusing on their perceptions regarding the threat

that pathogens carried on their own hands pose to the susceptible patient. To change the HCWs' behavior, the intervention relies on environmental cues to action (hand hygiene signs and hand cultures), reinforcing perceived benefits (using signs containing gain-framed messages), removing barriers (systematic distribution of personal hand sanitizer), and HCWs' perceived susceptibility and severity of the patient and their role in increasing their patients' risk (patient-focused signs and display of hand cultures).

Signs and the display of HCW hand cultures are environmental cues to action to remind workers to disinfect their hands. The messages each portrays will also be based on psychology and health communication theory to enhance the effect of the cue to action. Specifically, the signs will contain gain-framed messages that emphasize the benefits of proper hand hygiene (as opposed to the harms associated with poor compliance). Although this distinction may seem subtle, accumulating evidence suggests that gain-framed messages are more effective than loss-framed messages at promoting certain health behaviors such as hand hygiene.[33, 59-60] The signs will also be patient-focused based on recent research and our own pilot work.[34] Patient-focused messages are congruent with the HBM and reinforce patient susceptibility (in the case of HCWs). We are testing the frequency of changing the signs because individuals can become habituated to cues to action following repeated exposure; therefore, periodically changing the signs may maintain their saliency over time.[61-64] Besides a cue to action, displaying the hand cultures of HCWs draws from HCWs' perceived susceptibility of their patients. We will ask HCWs to place their hands on blood agar plates, grow the pathogens on the plates in a lab, and then display pictures of the HCWs' hand cultures (identifiable only to the HCW themselves) on their wards/units. Finally, in surveys of HCWs' perceptions of hand hygiene compliance, poor access to hand-sanitizing products is consistently endorsed as a major barrier to hand hygiene.[51, 65-66] To counter this barrier, we will systematically provide individual hand sanitizers and holders to make hand hygiene feasible from any location in the hospital.

<u>Testing and Evaluating Inventions in Infection Control</u>: Choosing the appropriate study design is critical when performing infection control studies. Mixed methods designs are essential to test the effectiveness of an intervention and to evaluate how and why a particular intervention may or may not be effective. Understanding the institutional and organizational context and processes are an important piece of developing and testing an intervention that can be implemented and spread in VHA.

<u>Cluster Randomized Trials (CRT)</u>: To empirically test an intervention, randomized control trials (RCTs) are the gold standard. However, RCTs are often infeasible in infection control studies because inventions must occur at the population-level, which can often only be stratified at the hospital or unit. In this case, multi-hospital CRTs are the best option.[67-68] Additionally, when CRTs are small in size with, the intervention and control groups can be matched prior to randomization on pre-intervention measures of the outcome of interest (i.e., hand hygiene compliance rates) to help reduce the effects of unmeasured confounders and thereby import higher internal validity and improved power.[69] This ensures population-level interventions such as hand hygiene improvement are adequately evaluated. Dr. Perencevich, co-I, has used

stratified randomization with pair matching in a 20-ICU CRT funded by AHRQ (BUGG Study, PI: Harris)

Qualitative Process Evaluation: Qualitative process evaluation is important to understand the context in which the trial is being implemented.[70] Conducting a process evaluation in parallel to a CRT allows investigators to gather data on likely or potential barriers and facilitators to the intervention as it progresses through the research-to-implementation pipeline.[71-73] In addition, purposefully sampling hospitals or units from the cluster trial enhances the investigators ability to understand how the invention does or does not work in different settings including variables such as general medicine/surgical wards and ICUs, different regions of the country, or the size of the hospital. By conducting a qualitative process evaluation alongside a CRT, the intervention will be more successful in overcoming the barriers to the implementation and spread of evidence-based interventions.[74] Unfortunately, no hand hygiene studies to our knowledge have included qualitative process evaluations of their interventions.

Preliminary Studies

Comparing Direct Hand Hygiene Observation to Product Usage (PI: Perencevich, IIR 09-099): In this study, we are validating product usage to direct observation. The study started in March 2011 and is on-going, involving 11 wards and ICUs at Baltimore VA, Iowa City VA, and Portland VA. Through the study, we have developed a protocol for direct observation by anonymous observers (see Operational Primary Outcome Section), observer training and validation, and a system for data collection and analysis . We have recorded over 14,000 hand hygiene opportunities to date. Our findings are consistent with other hand hygiene studies in finding a 32.9% compliance rate upon entry and a 50.8% rate on exit.[15]

Testing theoretically-based hand hygiene messages: (Reisinger, Vander Weg and Perencevich) We have begun to pilot test theoretically-based messages on hand hygiene posters. Four distinct signs were designed using constructs from health behavior and communication theories: personal vs. patient susceptibility,[34] gain vs. loss framing,[75] and perceived social norms.[76] In addition, the signs were placed at point-of-use near all hand hygiene dispensers on the wards/units to increase their potential as cues to action as described in the HBM.[77] In February 2012, the 4 signs were placed next to hand hygiene dispensers on 5 randomly chosen wards/ICUs. The remaining 6 control wards/ICUs did not have signs. 13,221 hand hygiene opportunities were observed pre-intervention and 915 opportunities have been observed to date in the pilot intervention period. Baseline entry compliance was 38.8% in control and 34% on sign wards. Exit compliance was 56.4% in control and 52.5% on sign wards. Rooms with point-of-use signs had a non-significant 11.68% increase in exit (p=0.44) compliance. Importantly, the sign with patient-focused and gain-framed language had the highest entry compliance 53% vs 29% for the other signs (p=0.042) and the highest exit compliance 79% vs 52% for the other signs (p=0.36). Although the signs led to minimal improvement in HH compliance overall, the gain-framed, patient-focused sign was associated with substantial increases in entry and exit compliance. This finding highlights the potential importance of the specific type of messaging strategy that is used. Therefore, after completing additional observations to verify the effect, we will test variations of gain-framed, patient-focused signs. Given positive findings from gain-

framed messages[33] and patient-focused messages[34] on hand hygiene signs, we expect using all gain-framed, patient-focused signs will increase the effect of cue-to-action signs overall.

VA-wide Survey on Hand Hygiene Practices: Drs. Reisinger and Perencevich, in collaboration with the Deputy Under Secretary of Health's Office, were responsible for the design and analysis of a national survey to examine the variation in hand hygiene practices among VHA facilities. This 51-item intranet survey covered three major areas of hand hygiene: 1) methods of measuring HCWs' hand hygiene compliance, 2) HCW-directed interventions to improve hand hygiene compliance, and 3) site-specific threshold of hand hygiene compliance. An expert panel of VHA infection prevention staff and researchers reviewed a preliminary draft of the questionnaire for content validity and clarity and provided suggested modifications, which were subsequently incorporated into a revised version of the survey. A memo was sent from the Deputy Undersecretary requesting that the person most familiar with hand hygiene practices at each VHA medical center complete the survey. 141 (100%) facilities returned the survey. A vast majority (98.6%) of facilities conduct direct observations of HCWs to measure hand hygiene compliance rates. Approximately one quarter monitor product usage and 2.9% use automated monitoring systems. Most facilities (78.0%) train observers individually. Notably, less than half (45.3%) validated the observation process at the onset and fewer (39.6%) continue to validate observations over time, which is a critique of direct observations conducted in the context of research studies as well.[17, 78-79] About one-quarter do not attempt to blind staff to hand hygiene observations, a practice which is likely to contribute to reactivity and a Hawthorne effect, thereby artificially inflating reported hand hygiene rates.[19-20] In addition, variation existed as to which points of care were considered hand hygiene opportunities. The three most frequently endorsed were room exit (70.9%), room entry (68.1%), and after removing gloves (59.6%). The proportion who follow the 5 Moments of Hand Hygiene[2] as recommended by the latest guidelines were: 1) 53.3% before touching a patient, 2) 47.5% before clean/aseptic procedure, 3) 46.8% after body fluid exposure, 4) 56.7% after touching a patient, and 5) 48.2% after touching patient surroundings. These findings demonstrate that many VHA facilities are not following standard recommendations for training observers and conducting observation of HCW hand hygiene practices and likely accounts for the discrepancy between compliance rates reported as part of performance measures and those documented in the literature.[54, 80]

Strategies for improving hand hygiene compliance that were endorsed by facilities included posters (97.2%), feedback (to executive leadership [98.6%], units/clinics [93.5%], one-on-one coaching to HCWs observed to be noncompliant [93.5%]), and improved access to hand hygiene products (e.g., 90.6% made individual hand sanitizer available to staff). 88.5% have mandatory education programs. The majority of the facilities (77.3%) set their hand hygiene compliance target between 90-100%.

This VA-wide survey reveals variation in hand hygiene practices, reflects commonly accepted practices in HH improvement, and identifies gaps in measuring and improving compliance. Facilities continue to set high goals for hand hygiene compliance rates even though a standardized compliance target does not exist, while the OIG report found facilities are not reaching their target.[22] These data contributed to our decisions regarding which hand hygiene

interventions to include in the study in order to systematically disentangle hand hygiene bundles and improve hand hygiene compliance. We chose signs and individual hand sanitizers as a means of evaluating and improving the effectiveness of strategies commonly used in VHA.

Systematic Literature Review of Hand Hygiene Interventions: In addition to the hand hygiene survey, we are completing a meta-analysis of the existing hand hygiene intervention literature for the VHA's response to the OIG report. The meta-analysis will be finalized at the end of June 2012. We have searched 5 unique databases for studies published from 2000 to 2012: 1) National Institute of Health PubMed; 2) PsychInfo; 3) The Cumulative Index to Nursing and Allied Health Literature (CINAHL); 4) The Cochrane Library; and 5) Scopus. The search strategy includes terms such as hand hygiene, handwashing, hand antisepsis, and hand disinfection to identify studies. We developed the search strategy by working with a trained research librarian from the UI's Hardin Health Sciences Library to translate the search terms into thesaurus terms or Medical Subject Headings (MeSH) terms. For the overall time period (2000-2012), we found 3,200 articles. For the pilot data in this proposal, we focused specifically on articles from 2011 to present to update the 2011 Cochrane Review.[25] From this time period (2011 to present), we found 439 articles based on the search criteria. The titles and abstracts for the 439 articles were then reviewed for inclusion and exclusion criteria, which reduced the number of articles to 14. From this set of 14 articles, study investigators, including Drs. Reisinger and Perencevich, read the full article and appraised them to determine whether they fully met inclusion criteria and a minimum level of quality. Inclusion criteria for the systematic review consisted of randomized control trials, controlled clinical trials, before and after quasi-experimental studies, and interrupted time series analyses studies limited to healthcare facilities. At least 2 investigators independently assessed each article and a third investigator resolved any discrepancies. Two data extractors recorded data from each selected study on data collection forms designed for the systematic review. Recorded data included study design (e.g., cluster randomized trial, pre-post design, time series), interventions, outcome, adjustment for potential confounders, and potential biases. We rated the study quality using standard methods.[26, 81]

Overall, 12 of the 14 articles assessed met inclusion criteria. All but one of the studies was a before and after quasi-experimental design. Two-thirds (n=8) of the articles had less than 5,000 hand hygiene observations and only 2 had more than 10,000. Four of the 12 studies were conducted in the United States, while a majority (n=6) were conducted in European countries. Most studies (n=11) were conducted in acute-care facilities in general medical wards and intensive care units (ICUs), although 3 were conducted throughout entire hospitals. Following the recommendations of many in the field, only one study tested a single intervention (signs) and the remainder included multiple intervention components. The majority (n=9) of the studies included 4 or more types of intervention strategies, with 2 studies including over 7. None of the studies attempted to disentangle the individual effect of the interventions. The most commonly bundled intervention components were signs (n=9), followed by education (n=9), audit and feedback (n=8), and increased access to hand hygiene products (n=8). The final meta-analysis has not been completed, but hand hygiene compliance rates in pre-intervention periods ranged from 6.5% to 54.3% and from 49.9% to 95.6% in the post-intervention period. The study with the greatest improvement (43.3% to 95.6%, p<0.001) included over 9 interventions.[82]

Implementation of Tele-ICU in VISN 23: Over the past year, Drs. Reisinger and Moeckli have conducted a qualitative formative evaluation of the implementation of Tele-ICU in VISN 23 (IIR 09-336, P. Cram, PI). Members of the research team have conducted pre- and early post-implementation focus groups, semi-structured interviews, and site observations at 5 ICUs and the Tele-ICU support center. Data have been fed back to the Tele-ICU support center to improve processes of care and the effectiveness of their support. A key finding has been the lack of knowledge on the part of the bedside staff regarding how to effectively utilize the Tele-ICU Support Center and incongruent expectations between bedside and Tele-ICU staff regarding roles and responsibilities in processes of care. The team has worked with the Tele-ICU Support Center to improve its education and communication strategies to the bedside ICUs it supports. The hand hygiene proposal builds off of the positive and productive working relationships the qualitative team has built with these facilities and their leadership.

Unique Contribution to Hand Hygiene Literature and VHA Infection Control: This study will advance the science of hand hygiene by using a strong research design to understand individual strategies and their combined effect within a larger intervention bundle. For over a decade, multimodal hand hygiene interventions have been the consensus in the field;[46, 48] however, research designs continue to be weak and no studies have attempted to disentangled multifaceted hand hygiene interventions. In addition, by conducting a parallel mixed methods design combining a cluster RCT and a qualitative process evaluation, we will be better positioned to implement and spread findings from the study because we will know the barriers and have worked through potential solutions for the field of infection control. Our close collaboration with operational partners in VA will assist in making this feasible.

Operational Partner Collaboration: This project was designed in close consultation with the VA program offices that play key roles in MRSA prevention. Operational Partners from Patient Care Services include: the National Infectious Disease Service (**NIDS**) and the MRSA / Multidrug-resistant Program Office (**MDRO**). Partners from the Office of Public Health include: the National Center for Occupational Health and Infection Control (**COHIC**) and the *Infection: Don't Pass It On* (**IDPIO**) Program. **VISN 23** (Director Murphy and Dr. Julius) will be a further operational partner for this proposal.

This proposal has been shaped by continued discussions with our partners over the past 16 months, including an in-person meeting on August 8, 2011 in Cincinnati with Gary Roselle, MD (Director, NIDS), Martin Evans, MD (Director, MDRO), and Lewis Radonovich, MD (Director, COHIC). In addition, the project will benefit from the participation of 10 VA hospital epidemiologists who have agreed to be study investigators.

Importantly, the investigators are already working with the operational partners. For example, Dr. Perencevich is the Senior Associate for Infection Control Studies in COHIC, and Drs. Reisinger and Perencevich have worked with COHIC, the NIDS, the Office of Quality and Safety, and the National Center for Patient Safety to develop a response to "Issue 1: IP

Practices in Patient Care Units/Areas" in the recent VA OIG report, *Evaluation of MRSA Prevention Practices in VHA Facilities*.

B. Significance

Excellence in hand hygiene protects veterans from MRSA and other HAIs and is a major patient safety goal. In one study, a multifaceted hand hygiene intervention decreased HAIs by 42% and overall incidence of MRSA from 2.16 to 0.93 episodes per 10,000 patient days ($p<0.001$).[12] However, few evidence-based interventions for improving hand hygiene exist and none are included in the current VA Required Hand Hygiene Practices Directive (2011-007). In addition, the VA Office of Inspector General (OIG) recently issued a report entitled, *Evaluation of Prevention Practices in Veterans Health Administration Facilities (11-03361-274)*. In the report, OIG recommended that intervention strategies be initiated "when hand hygiene performance falls below established thresholds" as required by The Joint Commission. In response to OIG's recommendation, the Office of the Deputy Under Secretary for Health for Operations and Management (DUSHOM) and the Office of the Deputy Under Secretary for Health for Policy and Services (DUSHPS), Office of Public Health requested that Drs. Reisinger and Perencevich design and analyze a VA-wide facility survey of hand hygiene practices. (See Preliminary Studies Section The same Offices also asked Drs. Reisinger, Perencevich, and Schweizer to complete a meta-analysis of hand hygiene interventions. (See Preliminary Studies Section The Under Secretary of Health stated in his response to the OIG report that the results of these studies would be used to inform a web-based hand hygiene toolkit maintained by the Office of Public Health, IDPIO. Findings from this proposed study will be directly disseminated through the hand hygiene toolkit. Drs. Reisinger and Vander Weg will develop the materials for dissemination which will include the most effective hand hygiene signs, detailed information about the "bundled" intervention and its overall effect, and recommendations for tailoring and implementing the intervention based on the Aim 2. <u>Thus, this project will lead to immediate impact by providing products for dissemination to improve quality of care.</u>

The effort to improve hand hygiene practices continues to expand in VHA. Multiple VHA offices are coordinating efforts to establish a national hand hygiene initiative. Our study team is collaborating with several programs within the Offices of Public Health (COHIC, IDPIO) and Patient Care Services (IDPO) to reach this objective. The efforts include participation on the National Hand Hygiene Workgroup, chaired by Dr. Perencevich with Dr. Reisinger as a committee member, Dr. Reisinger chairing the Workgroup's Hand Hygiene Intervention Subgroup, and a serving on the planning committee and presenting at the VA-wide Summit promoting hand hygiene practices.

In addition, Elaine Larson, PhD, Loreen Herwaldt, MD, and John Boyce, MD, all of whom are internationally-recognized experts in hand hygiene and infection control, have served as advisors in developing the hand hygiene bundle being tested in this study. They have graciously agreed to continue to serve on quarterly Advisory Board meetings over the duration of the study to review the progress of the project and to assist in implementation and dissemination decisions.

Finally, this proposal will benefit from an annual Implementation and Dissemination Advisory Committee that will include all mentioned operation partners, the Hand Hygiene Advisory Board members, and include two VHA implementation experts: Dr. Anne Sales, an implementation science expert and recent acting director of the Inpatient Evaluation Center, and Sanjay Saint, MD (Ann Arbor VAMC), an expert in the implementation of infection prevention interventions. John Jernigan, the Director of the Office of HAI Prevention Research and Evaluation of the Centers for Disease Control and Prevention (CDC)'s Division of Healthcare Quality Promotion (DHQP), will also join the Advisory Committee. Dr. Jernigan has been involved in advising VA's MRSA Initiative for over 5 years starting with Pittsburgh's pilot study and through the recent long-term care study in VA CLCs that determined that 58% of CLC residents are MRSA colonized. Our research team and the studies we are conducting are posed to shape VHA policy on improving hand hygiene compliance and decreasing MRSA and other HAIs among our veterans.

3.0 Objectives

The proposed study will use a parallel, mixed-methods design that will integrate qualitative research (Aim 2) with a cluster-randomized controlled trial (Aim 1).

<u>Specific Aims</u>: The two specific aims and associated hypotheses of the project include:

1) Identify combinations of hand hygiene intervention strategies that optimize hand hygiene compliance and that could form an evidence-based hand hygiene bundle for VHA implementation.

   <u>Hypothesis 1</u>: Combinations of interventions will increase compliance rates more than single interventions.

Aim 1 will entail a 30-month cluster-randomized controlled trial that will sequentially test three individual hand hygiene interventions to identify an optimal combination of interventions to increase hand hygiene compliance. The trial will be conducted in 59 hospital units in 10 VA hospitals in order to test the efficacy of individual and then sequentially added interventions to determine their incremental impact on hand hygiene compliance.

2) Identify institutional, organizational, ward/ICU, and individual level facilitators and barriers to implementing hand hygiene interventions.

   <u>Hypothesis 2</u>: Facilitators and barriers will pattern around contextual factors such as level of leadership support and organization of infection control programs.

Aim 2 will entail a qualitative process evaluation that includes site visits to purposefully selected sites, semi-structured interviews, and observations to examine barriers and facilitators to the interventions and develop contextual insight for implementing and scaling-up the intervention at additional sites as a national initiative.

4.0 Resources and Personnel

The central site will be the Iowa City VA Health Care System. Research staff at Iowa City will be responsible for patient data pulled from VA administrative datasets and compiling and securely storing all hand hygiene observation data for Aim 1. Iowa City research staff will be the only staff performing Aim 2 (qualitative process evaluation). For Aim 2, the Iowa City research staff will recruit VA personnel to participate in individual and group semi-structured interviews. They will also be responsible for the informed consent protocol and conduct all of the interviews.
The nine other participating sites will contribute to efforts of Aim1 of the study. Each site will be in charge of 1) conducting observations of potential HCW hand hygiene opportunities (Site Research Coordinator), 2) hanging up the hand hygiene signs (Site PI/Hospital Epidemiologist), 3) working with the site's ICP to distribute individual hand sanitizers (Site PI/Hospital Epidemiologist), and 4) obtaining and displaying HCWs hand cultures (Site PI/Hospital Epidemiologist).

There will be 10 sites included in this study and they are detailed in section 5.1, which includes table 1, "Site Selection."

Data analysts at the Iowa City VA will perform analysis and be responsible for data management. Only Iowa City VA staff will have access to the data.

5.0 Study Procedures

5.1 Study Design

Site Selection: Table 1 lists the 10 VA hospitals participating in the study. The table also indicates whether the site is in VISN 23 and whether Aim 2 will be conducted at the site. The sites were carefully selected to represent geographical variation—covering the Northeast to Northwest and Midwestern, Southern, and Western states. In addition, the hospitals represent small, medium, and large facilities within VA. Finally, this study is embedded in a larger set of integrated studies as part of a CREATE application focused on combating MRSA and other HAIs. One of the broader aims of these studies is to develop a VISN-level computer model to assist leadership in policy decision-making around the spread of MRSA and other HAIs. Therefore, we selected all three tertiary care hospitals in VISN 23 to ensure we have a rich set of data for a single VISN. The 6 sites for Aim 2 were selected to balance the need for depth of data in a single VISN (Iowa City, Minneapolis, and Omaha), geographic variation (Baltimore, Portland, Miami, and VISN 23), and successful collaboration on previous projects (Baltimore, Iowa City, Portland). All general medicine and surgical wards and ICUs will participate in the study. (Psychiatric wards were excluded due to restrictions regarding alcohol-based hand sanitizer.)

Table 1: Site Selection

Site	Number of ICUs	Number of Wards	Total Number of Units/Wards	VISN 23	Aim 2
Ann Arbor VA	2	4	6		
Baltimore VA	3	2	5		X
Boston VA	3	8	11		
Iowa City VA	1	2	3	X	X
Miami VA	3	3	6		X
Minneapolis VA	2	7	9	X	X
Omaha VA	1	2	3	X	X
Portland VA	1	2	3		X
San Antonio VA	3	5	8		
Salt Lake City VA	2	3	5		
Total	21	38	59		

Aim 1 involves a sequential cluster-randomized controlled trial that will be conducted in 10 VA hospitals to evaluate the individual and combined impact of three approaches to improving hand hygiene compliance. The primary outcome measure will be directly observed hand hygiene. The unit of analysis will be hospital wards/ICUs.

Outcome Measures**:** Hand hygiene compliance is the primary outcome measure. Compliance rates will be determined using the same methods of direct observation of HCWs developed by Dr. Perencevich for his VA HSR&D funded study (IIR 09-099). (See Cluster Randomized Trials (CRT) in 2.A.) The secondary outcome measure is MRSA acquisition. Both compliance and MRSA infection rates will be collected monthly throughout the project for each of the 59 units.

Operationalization of Primary Outcome: Nurses, physicians and other staff will be observed for 10 consecutive minutes of clinical activity on the study wards and ICUs. Observers will be research staff specifically trained and validated in hand hygiene observations. (See Appendix for standardized observation worksheet.) The observation periods will be 10 minutes long so that observers can be transiently on the wards/units without their presence being noticed, which should minimize the Hawthorne effect.[14] As Dr. Perencevich has done in his previous study, the observers will be given a "cover story" so that the true purpose of their observations will be hidden.[14] Therefore, while observers will be on the wards, the HCWs will not know they are being watched for infection control purposes, so they will not artificially increase their compliance. In ICU settings, we will state that the observers are collecting data for a severity of illness aggregate measure, such as the APACHE score. In non-ICU, medical/surgical ward settings the observer will state that they are doing a patient movement and activity of daily living survey to determine the proportion of time patients who are isolated or non-isolated receive their trays of food or are transported outside of their rooms to physical therapy or radiology, etc. The standardized observation instructions and training protocol has been successfully implemented in other studies by the team.[14-15] Hand hygiene opportunities at entry and exit of a patient's room will be recorded. We are using entry and exit because it is the one of the most common "hand hygiene opportunities" reported in the literature and by VA facilities. However, we will also record observations of the 5 Moments of Hand Hygiene as current hand hygiene observation guidelines recommend. [2, 54, 80] Observations will occur during the day shift (75%) and night shift (25%). In addition, observations will include weekends (20%) and weekdays (80%).

Operationalization of Secondary Outcome: MRSA acquisition is defined as patients who are MRSA– and become MRSA+. MRSA acquisition will be analyzed as a rate looking at number of acquisitions per patient days per month on a ward/unit level basis. Healthcare-associated MRSA infections are collected by the VA Inpatient Evaluation Center (IPEC) and are defined according to guidelines of the CDC's National Healthcare Safety Network (NHSN). IPEC provides facility-level information about MRSA prevalence on a monthly basis. Since 2007, MRSA/MDRO coordinators at individual facilities have entered monthly data on surveillance testing and on the prevalence and acquisition of MRSA as well as the frequency of specific types of clinical MRSA infections including skin and soft tissue infections, urinary tract infections and central-line associated bloodstream infections into an SQL database maintained on a server at IPEC.[5]

Interventions: Based on our systematic review, VA-wide survey, and our own pilot data we have identified the three interventions with the greatest likelihood of improving hand hygiene compliance and also in need of further study individually and in combination. The three interventions are: 1) theoretically-based and empirically-tested hand hygiene signs to serve as environmental cues to action and to reinforce of HCWs' perceptions of patient susceptibility, 2) personal hand sanitizers to decrease barriers and improve accessibility to hand sanitizer, and 3) HCW hand cultures to test the combination of individual feedback, environmental cues to action, and HCW perceptions of patient susceptibility and severity. This proposal will identify an optimal combination of interventions to increase and sustain hand hygiene compliance.

Varying Environmental Cues to Action (Hand Hygiene Signs): Hand hygiene signs and posters are one of the most common infection prevention interventions utilized in healthcare settings.[2, 24, 27] Among VA hospitals, 97.2% report displaying hand hygiene signs in their facilities. Signs are also a low cost, easy to implement intervention. Furthermore, signs act as environmental cues to action, which the Health Belief Model suggests are important in triggering targeted behaviors or actions.[58] However, signs are often quickly designed and taped up (e.g., "Foam In, Foam Out") or printed off of a website promoting hand hygiene. They are rarely theoretically-based or empirically-tested. Recent evidence suggests that the content of signage affects the degree to which it prompts hand hygiene behavior;[33-34] therefore, the specific message is an important component that needs to be considered. In preliminary studies, we have investigated the most effective theoretically-based messages to display on signs to improve HCW behavior. (See Preliminary Studies Section.) As discussed previously, gain-framed messages focused on helping patients appear to be most effective in changing HCW behavior. However, evidence also strongly suggests that repeated exposure to the same cues leads to a process of habituation such that their effectiveness at prompting behavior is diminished over time.[61-64] Therefore, it is important to identify strategies for maintaining the salience of hand hygiene cues. One approach is to periodically modify the cues to increase their novelty and enhance the potential that HCWs will attend to and act on them.[54] For the current proposal, we will study how to sustain their power as a cue to action by varying the frequency of changing the signs.

The specific signs that we will use in the proposed study will be based on those found to be most effective from our ongoing pilot work. We will use signs from the next phase of our pilot work in which we are testing multiple gain-framed, patient-focused signs with slight variations on message verbiage, color, and HCW and patient demographics. The six most effective signs will be changed at varying frequency depending on their randomization assignment. Although evidence suggests that periodically changing reminders is likely to reduce habituation and enhance their impact, little is known about the frequency at which this should occur. Therefore, signs will be changed at varying frequencies: every week, once a month, or displayed for the entire 6 months of this phase of the study, with effects on hand hygiene compliance compared across conditions. We will continue to use point-of-use to determine the placement of the signs. During the first site visits the qualitative team will document current practices surrounding the display of hand hygiene posters. They will also discuss how to best implement point-of-use signs with the infection control (IC) team at each site. We have worked with Baltimore VA, Iowa City VA, and Portland VA to create solutions to displaying signs next to dispensers on hospital units. For those sites at which we do not conduct site visits as part of Aim 2, we will conduct interviews over the phone to gather the necessary information. We will conduct the phone interviews after the site visits in ensure we will be able to integrate all necessary questions into the interview guide. The Site PI/Hospital Epidemiologist PI will be responsible for hanging the hand hygiene signs according to the protocol established for each site.

Distribution of Individual Hand Sanitizer Gel to HCWs: Easy access to hand hygiene products is known to improve hand hygiene compliance.[12, 51, 66, 83-84] The most common recommendation is to place alcohol-based hand rub dispensers at a patient's bedside. However, due to space configurations and fire codes, many hospitals are unable to implement this recommendation. To counter this barrier, some have recommended providing individual hand sanitizers to all healthcare workers.[85-87] In the VA national survey led by members of our investigative team, 90.6% of VA hospitals reported that they provide staff with individual containers of hand sanitizer; however, the frequency of disbursement is inconsistent. Providing hand sanitizer to staff can also take a variety of forms from placing a jar of individual dispensers at a nursing station to making them available at a staff meeting. (Personal communication, Chris Kerper, 11/10/11) During the site observations, we will document the current practices of the sites and contact the four sites at which we are not conducting a qualitative evaluation to obtain information regarding current practices and how strategies for providing personal hand sanitizer may be improved to maximize usage.

Figure 2. Image of Organisms Grown from HCW Handprint on a Blood Agar Culture Plate
Courtesy of John Boyce.

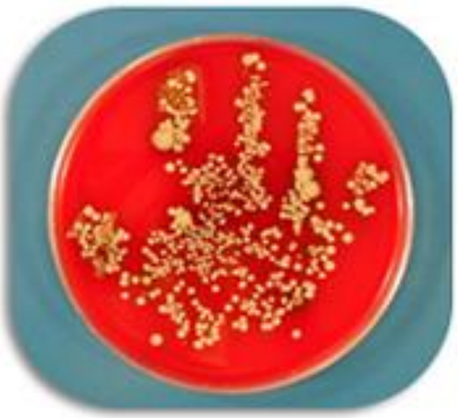

Bottles of individual hand sanitizer will be distributed to HCWs at the intervention hospitals. The Site PI/Hospital Epidemiologist will coordinate distribution with the Nurse Managers and Medical Directors overseeing the units. The Research Coordinator will document how the hand sanitizers were distributed at each facility and provide the documentation to the Iowa City Research Coordinator. As part of our hand hygiene observation protocol, we will also document the source of the hand sanitizer that is used (e.g., individual hand sanitizer, wall dispenser at point of care, etc.), as well as whether the individual hand sanitizer is visibly present on the HCW, to determine the extent to which this approach contributes to overall hand hygiene.

Displaying HCWs' Hand Cultures: Individualized feedback is an effective method of changing HCW behavior; however, it is a difficult strategy to implement when trying to improve hand hygiene compliance rates. Tracking hand hygiene compliance to an individual HCW through direct observation can be difficult because ideally the workers should be unaware they are being observed. Monitoring individual behavior without following a HCW's movement would be nearly impossible. Others have promoted automated hand hygiene monitoring systems as a means of tracking individual behavior without the barriers direct observation can pose. However, technical failures and lack of acceptance from staff impede the implementation of these systems.[20, 37, 88]

An innovative approach is to display the hand cultures of HCWs.[53-54] With this approach, HCWs are asked to place their hand on a blood agar plate in a large petri dish. The organisms left by the hand are then grown, a digital photograph is taken of the hand culture, and photographs of the cultures are displayed to provide feedback to the HCWs. (See Figure 2.) This combines the

effectiveness of individual feedback with environmental cues to action. Furthermore, direct visual evidence regarding the presence of pathogens on one's hands (and that of their colleagues) is likely to provide an especially salient affective or emotional source of motivation for improving hand hygiene (i.e., increases HCWs' sense of placing patients at risk).

For this intervention, the Site PI/Hospital Epidemiologist will be responsible for collecting blood agar culture plates of HCWs' hands on each unit once a month at the intervention hospitals. The Hospital Epidemiologist or a designated IC staff will go to each ward/unit and ask HCWs on the ward/unit at the time to place their hand on a blood agar plate. The HCW will be told the results (pictures of culture plates after organisms have been grown) will be confidentially displayed in staff work areas; however, if HCWs would like to their specific results, they can provide a four digit number that will be associated with their agar plate. We will not record their names or track the number. The number will be written on the culture plate and then on the digital photo for display. The research team will not record the number in any other way and will not record HCW names or any other identifying informations. HCWs will be responsible for their 4 digit number if they would like to find photo of their culture plate among the photos of culture plates from other HCWs. They will also be told the organisms left by their hands on the culture plates will not be identified (speciated), but any of the organisms grown on the culture plate could contribute to hospital-acquired infection in patients although likelihood varies by organism. Anyone on the ward/unit at the time will be eligible. HCWs will be able to opt out if they choose not to have their hand cultured. After organisms from HCWs hands are grown on the blood agar plates, a picture of the culture will be displayed in their unit with a four digit number attached (either provided by a HCW or randomly assigned). This will allow individual HCWs to confidentially view their own hands and the unit to see a general picture of the risk

Figure 3: Interventional Phases

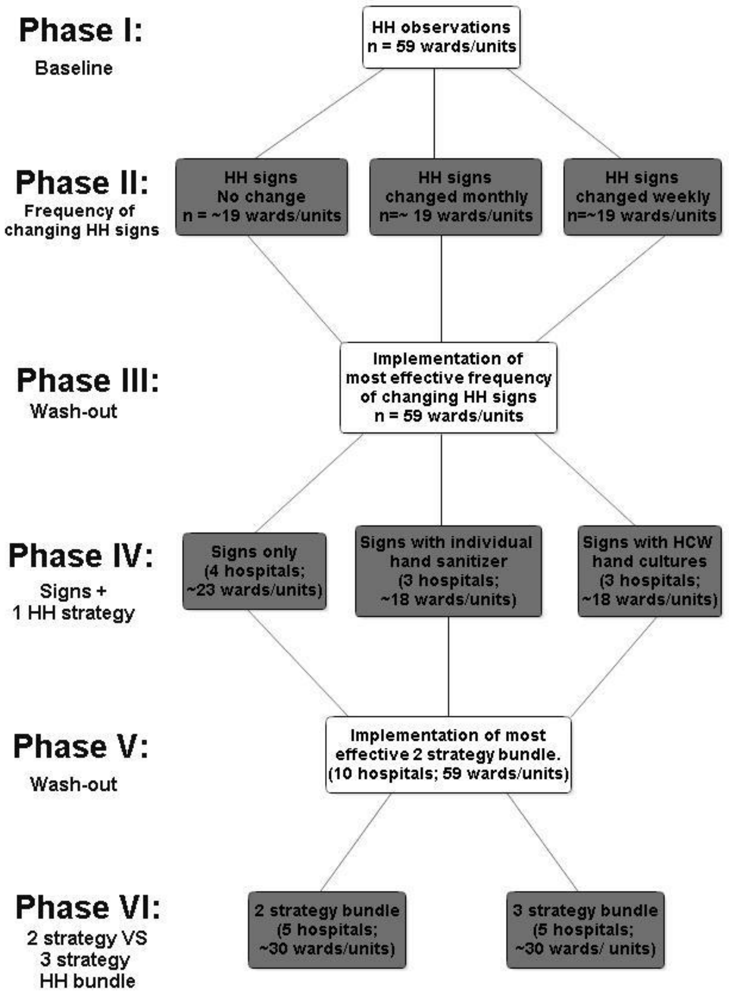

they pose to their patients. Photographs of the hand cultures will be displayed in common staff areas, but out of patient view. During the initial site visits, the qualitative team will observe current feedback mechanisms on the wards and units and work with the IC team to plan a way of displaying the feedback. This information will be used in interviews and planning sessions conducted with the four sites the qualitative team does not visit. Since the collected of culture plates is an intervention and not being done for data collection purposes, all culture plates will be destroyed via proper disposal techniques and digital photographs of the culture plates will be used for educational purposes only.

Study Procedure: The trial will entail 6 phases. (See Figure 3.) As an overview, Phases 2, 4 and 6 will be intervention periods and Phases 3 and 5 will establish a new baseline and serve as washout periods. Importantly, Phase 2 randomization will occur at the ward/ICU level since our pilot data suggests room entry signs can be randomized at the ward/ICU level. However, to avoid crossover effects, the other interventions will be randomized at the hospital level in Phases 4 and 6.

Phase 1 will obtain baseline rates of hand hygiene compliance and MRSA on each unit at each hospital. _Phase 2_ will compare different strategies for changing hand hygiene signage on hospital units to determine if periodically varying signage more effectively modifies hand hygiene behavior than using static environmental cues. The 59 individual hospital units will be randomized to 3 groups (~19 units per group): 1) no change in signs; 2) changing signs monthly; and 3) changing signs weekly. To improve power, we will use stratified randomization with matching based on baseline hand hygiene compliance rates during Months 1-6.[69] Block randomization will be used to ensure that baseline compliance rates will be evenly distributed across conditions. To do this, we will rank the units by compliance rate and then randomize by blocks of three using a computer-generated list of random numbers prepared by the Data Manger in consultation with the biostatistician. During _Phase 3_, the sign change strategy that was associated with the highest hand hygiene compliance will be universally implemented in the 59 study units in all 10 hospitals, this will set a new baseline and serve as a wash-out period. In our current study of hand hygiene interventions, we were able to detect a difference within six weeks. Thus, 3 months is likely enough time for wash-out.

Phase 4 will compare the additional benefits of the other 2 interventions–distribution of individual hand sanitizers to healthcare workers and obtaining cultures of healthcare workers' hands with visual feedback on hand contamination. In Phase 4, hospitals will be randomized to 3 groups: 1) signage only (4 hospitals); 2) signage and hand sanitizers (3 hospitals); and 3) signage and hand cultures (3 hospitals). Group assignment will again be stratified using block randomization (4, 3, 3), based on hand hygiene compliance rates measured during Phase 3. Individual strategies will be randomized by hospital to decrease contamination between wards/units. During _Phase 5_, the Phase 4 intervention associated with the highest compliance rates will be universally implemented; if both interventions were equally efficacious, the signage only hospitals will be randomized to receive either the hand sanitizer or hand culture intervention. Phase 5, like phase 3 will also serve as a washout period. _Phase 6_ will determine the potential incremental benefits of all 3 interventions in combination by re-randomizing

hospitals to 2 groups: 1) signage and one of the other interventions (5 hospitals); and 2) all 3 interventions (5 hospitals). Again, the interventions will be randomized by hospital to decrease likelihood of contamination.

Given that signs are already a frequently utilized, well-accepted, inexpensive, and easy to implement strategy, we are starting the intervention with theoretically-based, empirically-supported signs and testing the rate at which they should be changed in order to most effectively enhance HCW hand hygiene. Individual hand sanitizers are also relatively low cost and are currently available to HCWs in most VA hospitals. However, little is known about their impact on improving hand hygiene compliance rates as a stand-alone intervention. Finally, displaying the results of HCWs' blood culture plates is an innovative new strategy that has developed primarily in quality improvement projects, but it has never been directly tested against (or in combination with) other interventions. It is also relatively low cost at $1 per plate and involve little lab processing. Collectively, this approach will allow us to evaluate the individual and combined effects of the different intervention strategies in order to identify the optimal hand hygiene bundle.

Aim 2 is a qualitative process evaluation at 6 sites in the study. It involves pre-intervention and post-intervention site visits by the PI and Qualitative Analyst, which will include observations, semi-structured interviews with the IC team, and focus groups with ward/ICU staff. Semi-structured phone interviews will also be conducted with the IC team after each intervention is implemented.

During the first 6 months (prior to the baseline period [Phase 1]), Drs. Reisinger and Moeckli will travel to each of the 6 hospitals selected for Aim 2 and oversee a qualitative process evaluation of hand hygiene practices. The evaluation will include observations on individual units, semi-structured interviews with IC staff and leadership, and focus groups with staff on one high and one low performing unit at each hospital. Semi-structured interviews will be conducted individually with the IC team at each site, as well as site leadership. (See Appendix for Interview Guide.) The IC team includes the hospital epidemiologist, Infection Control Professional (ICP), and MRSA/MDRO Coordinator. Leadership will include those who oversee the IC team and nurse managers of the high and low performing units. The questions will focus on the history of infection control at the hospital (e.g., changes in personnel, gaps in staffing IC positions, integration of the MRSA Program, etc.), descriptions of how hand hygiene data are collected, goals for hand hygiene compliance, current interventions to promote hand hygiene, and plans to meet Joint Commission's National Patient Safety Goal for hand hygiene. The qualitative research team will also go on infection control rounds with the IC team with particular focus on the wards/units included in the study. The qualitative team will ask the IC team to describe what they look for during infection control rounds and identify barriers and facilitators to IC on each unit. The qualitative team will then return to those units to record hand hygiene specific data such as number and placement of hand hygiene dispensers, number and placement of hand hygiene signs, and whether and how hand hygiene compliance data are displayed on the unit and/or reported back to the staff. Field notes from both observation periods will be recorded.

The qualitative team will then conduct focus groups with the lowest and highest performing units at the hospital. (See Appendix for Focus Group Guide.) The focus groups will cover questions on knowledge of hand hygiene guidelines, the unit's interactions with IC, and current and past interventions to try to improve compliance. The qualitative team will return to Iowa City to analyze the data and develop case studies of each hospital's hand hygiene practices, including compliance data collected for the study. This will serve as a baseline description of the "hand hygiene culture" of each facility.

After each interventional phase is implemented, semi-structured interviews will be conducted over the phone with the IC team and nurse managers at each hospital (during the washout periods and during the post-intervention qualitative evaluation). The interviews will focus on perceived barriers and facilitators to implementation, staff reaction to the strategies, perceived impact on workflow and patient/provider relationships, and perceptions of each intervention's fit with the culture of the hospital.

Toward the end of Phase 6, Drs. Reisinger and Moeckli will return to the sites and replicate the same qualitative evaluation protocol. The qualitative evaluation will identify changes in "hand hygiene cultures" and document feedback about the interventions from frontline staff during unit focus groups.

All interviews will be audiorecorded on digital encrypted recorders or over the phone directly to a secure server. They will then be transcribed by in-house transcribers and reviewed for accuracy. All textual data (field notes and transcripts) will then be imported into MAXQDA, a qualitative data management and analysis software program.[91]

Risk protection

AIM I:
With regards to observations of hand hygiene compliance, no individual identifying information will be collected from healthcare workers or patients. Data regarding MRSA and hospital-acquired infection rates will be collected from the VA database, IPEC. All data will be collected anonymously. There is no patient-level data for the observation portion of the study; healthcare worker data will be anonymously observed and then aggregated. Removal of access to research study data will be accomplished for study personnel when they are no longer part of the research team and any incidents involving theft of data, etc., will be reported immediately. MRSA and hospital-acquired infection outcome data will be analyzed using VA datasets. Given the number of veterans included in this retrospective analysis and the use of existing data, informed consent will not be obtained from patients. This practice is consistent with practices for studies that use existing administrative data and medical records data. However, our data management protocol will ensure the protection of the protected health information thereby minimizing risks to individual veterans. A waiver of consent is included.

Risk will be minimized by limiting access to data, maintaining study paper files in locked offices, and storing data on password-protected computers and on servers, which are secured in locked

rooms by the Iowa City VA Medical Center Office of Information Resources Management. The servers can only be accessed by individuals with OIT-created network accounts. Data will be secured using network directory permissions assigned by OIT at the direction of the PI; thus ensuring that only study personnel with the approval of the PI (per IRB requirements) have access to identifiable human subject data. Data collection, management, and analyses will be compliant with VA data use agreements. In addition, reports of study findings will not identify individual HCWs.

The servers can only be accessed by individuals with IRM-created network accounts. Data will be secured using network directory permissions assigned by IRM at the direction of the PI; thus ensuring that only study personnel with the approval of the PI (per IRB requirements) have access to identifiable human subject data. Data collection, management, and analyses will be compliant with VA data use agreements. In addition, reports of study findings will not identify individual patients.

AIM 2:

VA Infection Control (IC) Team and Leadership: For six designated sites, a site visit will be coordinated with the Site PI assistance. Each member of the IC Team will be emailed a letter of invitation from the PI of the proposed research project. The letter will contain elements of consent. The PI or a member of her study team will then review the letter before conducting an interview with any member of the IC team. Site visits will take place during the baseline period and at the end of the interventions.

Four designated sites will not participate in site visits, but will participate in phone interviews with at baseline and post-intervention. Each member of the IC Team will be emailed a letter of invitation from the PI of the proposed research project. The letter will contain elements of consent. The PI or a member of her study team will then review the letter on the phone before conducting an interview with any member of the IC team.

In addition, phone interviews will be conducted at all 10 sites during the washout periods to gather any feedback from the IC team on implementation barriers for the previous intervention conducted at their site.

VA Healthcare Workers (HCWs): With assistance from the Site PI, the Iowa City Research Coordinator will contact the appropriate supervisors and inform them of the Qualitative Team's site visit. She will arrange a time for the focus group on two different units. At the time of the focus group, participants will be given an informational sheet and the PI will review the elements of consent (see focus group script with elements of consent and information sheet) with the participants in the focus group. They will be given the opportunity to ask questions and have all their questions answered. Participation in the focus group will be an indication of consent. This consent process has worked well in other focus group projects the team as conducted.

Data Collection: The study personnel will be responsible for reviewing elements of consent before each point of data collection.

Interviews and focus groups will be recorded on encrypted recorders and files will be uploaded directly to a secure server. Recordings will be transcribed and reviewed for accuracy. All data (recordings, transcripts) will be stored on a secured server. Each participant will be assigned a unique study identifier which will be used on all data. Subject identities (e.g., names, addresses, professional title) will be kept separate from the data and linkage information will only be available to the PI and Research Coordinator.

For proposed research, confidentiality risk will be minimized by limiting access to data to study personnel, removing unique participant identifiers (e.g., names) from analytical files, maintaining study data forms and files in locked offices, and storing study databases on password-protected computer files housed on a secure server. This server is configured and maintained by VA IRM staff and meets all VA security standards. The server is in a secure location at the Iowa City VAMC, where it is in a video-monitored area behind locked doors. All analyses will be performed on data in which unique patient information has been replaced with study identification numbers to protect confidentiality. A list matching names and identification numbers will be stored in the secure server. The crosswalk data file will be accessible only to the PI and Research Coordinator.

The proposed research project does not involve the collection of biological specimens.

5.2 Recruitment Methods

AIM I:

All veterans admitted to acute care at the 10 VA medical centers during the study period will be included in this retrospective data analysis. Approximately 135,852 veterans will be included regardless of age, race, gender, or underlying health status. Additionally, women and minorities will be included in this study.

The hand hygiene behavior of all facility staff working on the wards/ICUs during the periods of hand hygiene observation will be recorded.

This study aim will not use any recruitment materials nor will subjects be paid.

AIM 2:

VA Infection Control (IC) Teams: Approximately 18hospital epidemiologists, Infection Control Professionals, and MRSA/MDRO Coordinators will be asked to participate in the study through in-person and phone semi-structured interviews (3 members of the IC team from 6 sites). Hospital epidemiologists, Infection Control Professionals, and MRSA/MDRO Coordinators from 4 additional sites will be asked to participate in the study through phone interviews (approximately 12 study participants, 3 members of the IC team from 4 sites). The hospital epidemiologist is a Site PI on the study and will facilitate introductions to the IC team. The IC team will be asked to participate because of the unique knowledge they have regarding how hand hygiene is currently monitored and promoted at each VA facility and may have unique

insights into the barriers and facilitators of implementation. The participants may not be veterans, but are important to the study due to role as VA practitioners.

VA Healthcare Workers (HCWs): Approximately 140-150 healthcare workers (facility, leadership, nurses, physicians, respiratory therapists, dieticians, etc.) will be asked to participate in the study through in-person individual and group interviews. Approximately 24 will be from facility leadership. We anticipate approximately 6 participants per focus group. Two focus groups will be conducted on different units within each of 6 VA facilities. Healthcare workers may or may not be veterans; however, their participation in the study is important as they will provide crucial insight into how hand hygiene is practiced at their facility and barriers and facilitators to the interventions being implemented in the study.

We will use email letters and informational sheets to assist in recruiting HCWs for individual and semi-structured interviews and focus groups for Aim 2. Participants will not be paid.

5.3 Informed Consent Procedures

We will request a waiver of informed consent for all parts of the study except the semi-structured interviews (Aim 2). For Aim 2, we will request waiver of documentation of informed consent.

Aim 2 consists of HCW semi-structured interviews. All participants will receive information in both oral and/or written form regarding the risk and benefits of the project, confidentiality procedures, and study personnel to contact regarding questions and concerns (including contact numbers for the Central IRB). The PI or members of the study team will be responsible for reviewing elements of consent before each point of data collection. Participants will be given the opportunity to ask questions and have all their questions answered. Participation in the interview will be an indication of consent.

All interviews will be conducted by the Iowa City Qualitative Team and consent procedures will be conducted by Dr. Heather Reisinger or the Iowa City study team. No investigators/research assistants from local sites will be performing interviews or consent procedures.

5.4 Inclusion/Exclusion Criteria

AIM 1:

All veterans admitted to acute care at the 10 VA medical centers during the study period will be included in this retrospective data analysis. Approximately 135,852 veterans will be included regardless of age, race, gender, or underlying health status. Additionally, women and minorities will be included in this study.

The hand hygiene behavior of all facility staff working on the wards/ICUs during the periods of hand hygiene observation will be recorded.

AIM 2:

VA Infection Control (IC) Teams: Approximately 50 hospital epidemiologists, Infection Control Professionals, and MRSA/MDRO Coordinators will be asked to participate in the study through in-person and phone semi-structured interviews (3 members of the IC team from 6 sites). Hospital epidemiologists, Infection Control Professionals, and MRSA/MDRO Coordinators from 4 additional sites will be asked to participate in the study through phone interviews. The participants may not be veterans, but are important to the study due to role as VA practitioners.

VA Healthcare Workers (HCWs): Approximately 150 healthcare workers (facility, leadership, nurses, physicians, respiratory therapists, dieticians, etc.) will be asked to participate in the study through in-person individual and group interviews. We anticipate approximately 6 participants per focus group. Two focus groups will be conducted on different units within each of 6 VA facilities. Healthcare workers may or may not be veterans.

5.5 Study Evaluations

Participants will not be required to complete any procedures as part of the study. The only evaluation that some participants will participate in are semi-structured interviews among VA staff.

5.6 Data Analysis

AIM 1:

*Sample Size***:** We will collect continuous baseline data from each unit during the 6 month period prior to intervention. We estimate that 10-14 observations/hour can be witnessed based on our previous studies.[14, 89] Taking the lower estimate of 10/hour and assuming 60 hours/month of observation per site for a total of 600 hours/month projects to 36,000 observations during the 6 months pre-intervention period. (See Project Gantt Chart**.**) These will be spaced by unit and day/night shift as mentioned above. Given the 59 wards/ICUs, we will have an average of 120 directly observed hand hygiene episodes in each unit/month.

Power Calculation: Using our primary outcome, we estimated hand hygiene rates for each phase of the study. Given 30,000 observations in the group randomized to the intervention and in the group randomized to the control, each phase of the study will have 99% power to detect a 5% increase in hand hygiene rates due to the intervention (e.g., 60% to 65%). (See Table 2.)

Table 2. Power Calculations for Each Phase of Study

	Baseline Hand Hygiene Rate	Power to Detect a 5% Increase in Hand Hygiene Rate
Phase 2	60%	99%
Phase 4	65%	99%
Phase 6	70%	99%

Analysis: Following methods we have outlined previously,[90] we will use quasi-Poisson mixed effects models to account for matching and the cluster-randomization design, as observations over time within individual rooms, units and hospitals are expected to be correlated. The

dependent variable in each model will be the monthly hand hygiene compliance rate. The numerator will be the number of times a HCW disinfects their hands while the denominator will be the number of opportunities for hand hygiene. The ideal denominator is the WHO 5 Moments of Hand Hygiene [2] and where possible, we will collect these data. However, our current IIR 09-099 suggests that the most reliably measurable moments are room entry and room exit. Separate models will be run for the WHO 5 moments (combined as one dichotomous measure and for each of the individual "moments"), room entry compliance, and room exit compliance. Independent variables will include the tested intervention, the bed size and type of unit, monthly census, estimated monthly nurse to patient ratios, and other known confounders associated with hand hygiene compliance. The results will advance understanding of the effectiveness of individual interventions and their combined effectiveness as a bundle.

<u>AIM 2</u>:

<u>Analysis</u>: After the initial site visits, the study team will review transcripts and conduct an initial thematic content analysis.[92-93] Drs. Reisinger, Moeckli and Ms. Páez will read a subset of transcripts and generate a preliminary codebook through a series of meetings. The thematic codes will include *a priori* codes such as hand hygiene compliance data collection, hand hygiene interventions, facilitators and barriers to hand hygiene compliance, as well as inductive codes which are likely to emerge during data collection. The codebook will consist of top-level thematic codes, which will be used to "tag" textual data with the main themes.[94] The codebook will then be tested against subsequent interviews.

To test the codebook, Dr. Reisinger and the coding team will code one IC team member interview and one staff focus group and enter coding in MAXQDA10 individually. We will then meet together to review results. MAXQDA10 allows for side-by-side comparison of coding and a measure of agreement. We will systematically compare coding and discuss coding until consensus is reached. Final consensus will be entered in MAXQDA10. If measure of agreement is 80% or higher between coders, the coding team will divide the transcripts and will continue to code the remainder of the focus groups and interviews, periodically coding the same transcript to test whether agreement continues at the 80% level. If the agreement is not 80% for all codes, Dr. Reisinger will continue to work with the team and revisions to the codebook will be discussed. The process will be repeated until 80% agreement is reached. At least 10% of the transcripts will be consensus coded during the coding period.

During Phases 2-6, the qualitative team will continue to apply the codebook to the newly conducted interviews, but it is likely the codebook will expand to include specific codes about the study interventions. The coding team will continue to test agreement as the codebook is revised.

In the final Phase, the coding team will code the transcripts from the post-intervention site visits. The analysis will assist in writing a second case study of each hospital. The revised case studies will highlight changes in the "hand hygiene culture". It will also be used to develop a report on the facilitators and barriers to the interventions within the context of the intervention

sites. This report will be disseminated with the intervention results to improve implementation of the final hand hygiene bundle.

After top-level coding is complete, the research team will discuss which codes warrant more detailed subcoding. Our team has followed Miles and Huberman's[95] approach to subcoding, called matrix analysis, in several projects. In matrix coding, we develop a list of subcodes under a top-level theme and then code both for the presence or absence of the subcode, as well as content. MAXQDA10 has several features which aid in this level of analysis, including hierarchical coding, weighting of coded segments, and multi-factor queries in matrices. This coding will provide more detailed analysis on specific issues the research team finds most relevant and will primarily be used in the development of manuscripts for peer-reviewed publications.

Project Gantt Chart

	Qualitative Evaluation 1-6	Phase1 Months 7-12	Phase 2 Months 13-18	Phase 3 Months 19-21	Phase 4 Months 22-27	Phase 5 Months 28-30	Phase 6 Months 31-36	Qualitative Evaluation 37-42
HH Observations								
	**Aim 1**							
Randomization			-Control: no change in signs<br>-Arm 1: signs changed monthly<br>-Arm 2: signs changed weekly	-Implementation of most effective sign change frequency	-Control: Signs only<br>-Arm C: Signs and pocket hand sanitizer<br>-Arm D: Signs and HCW hand cultures	-Arm C only<br>**OR**<br>-Arm D only	-Arm C only<br>**OR**<br>-Arm D only<br>**AND**<br>-Arm E: Signs, pocket hand sanitizers, and HCW hand cultures	
Analysis								
	**Aim 2**							
Site Visits								
Semi-structured interviews								
Analysis								
	**Dissemination and Implementation**							
Quarterly Advisory Panel Meetings								
Annual Meetings with CREATE Dissemination/ Implementation Committee								
Submit manuscripts to								

peer-reviewed journals								

5.7 Withdrawal of Subjects

We do not anticipate any circumstances under which subjects would be withdrawn from research without their consent.

Subjects are entitled to decide to end their participation during an interview without any loss of benefits to which they are entitled. Their interview will be ended. If the participant would like to withdraw after the interview has already occurred, for data collected prior to withdrawal, the study team may continue to review the data already collected for the study but cannot collect further information, except from public records.

6.0 Reporting

Since the study does not involve any interventions/procedures with patients, we anticipate that adverse events will not occur at any sites. (In this study an SAE will not include diagnosis or treatment of MRSA – infections are expected to continue and will therefore not be reported to VA Central IRB or considered Unanticipated SAEs or UAPs).

Reporting of Events to the VA Central IRB: Serious Adverse Events and Serious Unanticipated Problems involving risks to participants and others will be reported to the VA Central IRB. This is a minimal risk study that does not involve a medical intervention. Protocol violations due to human error might occur in the form of breaches to confidentiality and privacy. However, any SAE, serious unanticipated problem, and/or protocol violation occurring during the course of the study will be collected, documented, and reported by the Local Site Investigator to the VA Central IRB (after consultation and review with the PI and Co-PI) within the required time frame (5 business days after the study is made aware of the occurrence). The project coordinator will review Adverse Event Forms with the PI and Co- PI to determine remedies that might serve to prevent future occurrences and to determine if it meets criteria for a SAE or serious unanticipated problem or a reportable protocol deviation. The project coordinator will follow all adverse events to the point of a satisfactory resolution.

7.0 Privacy and Confidentiality

AIM 1:

The anonymous data from the observations will be saved on secure servers is behind the VA firewall, only accessible from the VA network, at the collaborating sites and the Iowa City VAMC.

Data will be obtained from patient-level databases available through the Austin Automation Center, the VA Corporate Data Warehouse, electronic medical records available through the VA Computerized Patient Record System (CPRS), and VINCI. Only data already recorded in the medical records will be subject to recording and analysis. This research will not involve any contact with human subjects; therefore, no data outside of what is in the existing medical records will be collected.

AIM 2:

Interviews will be recorded on encrypted digital recorders and downloaded directly onto a VA server. Phone interviews will be recorded directly onto the VA secured server. The study database is located on a server behind the VA firewall. The database folder is only accessible to IRB approved research team members. The database is also password protected. CADRE project data will be stored on the CADRE server, which is maintained, backed-up, and secured in a locked server room by the Iowa City VA's Office of Information Technology (OI&TOI&T) department. The server can only be accessed by individuals with an OI&T-created network account. Data are further secured using network directory permissions assigned by OI&T at the direction of the Principal Investigator (PI). This ensures that only study personnel with the approval of the PI (in accordance with IRB requirements) have access to identifiable human subject/patient data. Additionally, files on the network that contain identifiable human subject data are password protected, and individuals accessing the server on a client machine are instructed to password protect their screen saver in order to maintain data security. All data entry and analysis will occur on VA owned servers and desktop computers. No electronic study data will be stored on removable media of any kind.

The PI, Study Coordinator at Iowa City and Data Analysts will have access to PHI. The local site study staff will not have access to PHI (beyond what they always have in their roles as physicians/hospital epidemiologists).

8.0 Communication Plan

All local sites will be engaged in research. All local sites will complete appropriate documentation to obtain approval of Central IRB with the help of the Iowa City study staff. Study coordinators from all sites will participate in weekly or bi-weekly conference calls in order to stay informed of changes to protocol. These meetings will also serve to update local sites about the event of any Serious Adverse Events, Unanticipated problems, or interim results that may impact the conduct of the study.

In addition to meetings in which we discuss study procedures, site visits by Iowa City study staff will occur at certain intervals during the study, at which time adherence to IRB-approved protocol will be verified.

When the study reaches a point at which a local site is no longer needed to be engaged in research, the Iowa City study staff coordinator will notify local facility directors and local site investigators by telephone.

9.0 References

1. Boyce JM, Pittet D. Guideline for Hand Hygiene in Health-Care Settings. Recommendations of the Healthcare Infection Control Practices Advisory Committee and the HICPAC/SHEA/APIC/IDSA Hand Hygiene Task Force. Society for Healthcare Epidemiology of America/Association for Professionals in Infection Control/Infectious Diseases Society of America. *MMWR Recomm Rep.* 2002;51(RR-16):1-45, quiz CE41-44.

2. World Health O. *WHO Guidelines on Hand Hygiene in Health Care: First Global Patient Safety Challenge, Clean Care is Safer Care*. Geneva, Switzerland: WHO Press; 2009.

3. Erasmus V, Daha TJ, Brug H, et al. Systematic review of studies on compliance with hand hygiene guidelines in hospital care. *Infection control and hospital epidemiology : the official journal of the Society of Hospital Epidemiologists of America.* 2010;31(3):283-294.

4. Klevens RM, Morrison MA, Nadle J, et al. Invasive methicillin-resistant Staphylococcus aureus infections in the United States. *JAMA.* 2007;298(15):1763-1771.

5. Jain R, Kralovic SM, Evans ME, et al. Veterans Affairs initiative to prevent methicillin-resistant Staphylococcus aureus infections. *N Engl J Med.* 2011;364(15):1419-1430.

6. Boyce JM, Larson EL, Pittet D. Foreword. *Am J Infect Control.* May 2012;40(4 Suppl 1):S2.

7. Larson EL, Bryan JL, Adler LM, Blane C. A multifaceted approach to changing handwashing behavior. *American Journal of Infection Control.* 1997;25(1):3-10.

8. Backman C, Zoutman DE, Marck PB. An integrative review of the current evidence on the relationship between hand hygiene interventions and the incidence of health care-associated infections. *Am J Infect Control.* Jun 2008;36(5):333-348.

9. Aboelela SW, Stone PW, Larson EL. Effectiveness of bundled behavioural interventions to control healthcare-associated infections: a systematic review of the literature. *The Journal of hospital infection.* 2007;66(2):101-108.

10. Larson E. Skin hygiene and infection prevention: more of the same or different approaches? *Clinical infectious diseases : an official publication of the Infectious Diseases Society of America.* 1999;29(5):1287-1294.

11. Larson EL, Early E, Cloonan P, Sugrue S, Parides M. An organizational climate intervention associated with increased handwashing and decreased nosocomial infections. *Behavioral medicine (Washington, D.C.).* 2000;26(1):14-22.

12. Pittet D, Hugonnet S, Harbarth S, et al. Effectiveness of a hospital-wide programme to improve compliance with hand hygiene. Infection Control Programme. *Lancet.* 2000;356(9238):1307-1312.

13. Pittet D. Compliance with hand disinfection and its impact on hospital-acquired infections. *The Journal of hospital infection.* 2001;48 Suppl A(Journal Article):S40-46.

14. Kim PW, Roghmann MC, Perencevich EN, Harris AD. Rates of hand disinfection associated with glove use, patient isolation, and changes between exposure to various body sites. *Am J Infect Control.* Apr 2003;31(2):97-103.

15. Morgan DJ, Pineles L, Shardell M, et al. The Effect Of Contact Precautions on Healthcare Worker Activity In Acute Care Hospitals. *Infection Control and Hospital Epidemiology.*(in press).

16. Reisinger HS, Yin J, Radonovich L, Hodgson M, Perencevich EN. Comprehensive Survey of Hand Hygiene Practices in the Veterans Health Administration. *IDWeek 2012: Advancing Science, Improving Care*. San Diego, CA, 2005.

17. Dhar S, Tansek R, Toftey EA, et al. Observer bias in hand hygiene compliance reporting. *Infect Control Hosp Epidemiol.* Aug 2010;31(8):869-870.

18. The Joint Commission. Measuring Hand Hygiene Adherence: Overcoming the Challenges. 2009.

19. Eckmanns T, Bessert J, Behnke M, Gastmeier P, Ruden H. Compliance with antiseptic hand rub use in intensive care units: the Hawthorne effect. *Infect Control Hosp Epidemiol.* Sep 2006;27(9):931-934.

20. Ellingson K, Polgreen PM, Schneider A, et al. Healthcare personnel perceptions of hand hygiene monitoring technology. *Infect Control Hosp Epidemiol.* Nov 2011;32(11):1091-1096.

21. Chassin MR. An interview with Mark Chassin. Interview by Steven Berman. *Jt Comm J Qual Patient Saf.* Oct 2010;36(10):475-479.

22. Office of Inspector General. Combined Assessment Program Summary Report: Evaluation of Infection Prevention Practices in Veterans Health Administration Facilities. In: Department of Veterans Affairs, ed2011.

23. Son C, Chuck T, Childers T, et al. Practically speaking: rethinking hand hygiene improvement programs in health care settings. *Am J Infect Control.* Nov 2011;39(9):716-724.

24. Naikoba S, Hayward A. The effectiveness of interventions aimed at increasing handwashing in healthcare workers - a systematic review. *The Journal of hospital infection.* 2001;47(3):173-180.

25. Gould DJ, Moralejo D, Drey N, Chudleigh JH. Interventions to improve hand hygiene compliance in patient care. *Cochrane database of systematic reviews (Online).* 2010;(9)(9):CD005186.

26. Larson E. State-of-the-science--2004: time for a "No Excuses/No Tolerance" (NET) strategy. *Am J Infect Control.* Nov 2005;33(9):548-557.

27. Pincock T, Bernstein P, Warthman S, Holst E. Bundling hand hygiene interventions and measurement to decrease health care-associated infections. *Am J Infect Control.* May 2012;40(4 Suppl 1):S18-27.

28. Gould D, Chamberlain A. The use of a ward-based educational teaching package to enhance nurses' compliance with infection control procedures. *Journal of Clinical Nursing.* 1997;6(1):55-67.

29. Huang J, Jiang D, Wang X, et al. Changing knowledge, behavior, and practice related to universal precautions among hospital nurses in China. *Journal of continuing education in nursing.* 2002;33(5):217-224.

30. Helder OK, Brug J, Looman CW, van Goudoever JB, Kornelisse RF. The impact of an education program on hand hygiene compliance and nosocomial infection incidence in an urban neonatal intensive care unit: an intervention study with before and after comparison. *Int J Nurs Stud.* Oct 2010;47(10):1245-1252.

31. Nevo I, Fitzpatrick M, Thomas RE, et al. The efficacy of visual cues to improve hand hygiene compliance. *Simul Healthc.* Dec 2010;5(6):325-331.

32. Thomas M, Gillespie W, Krauss J, et al. Focus group data as a tool in assessing effectiveness of a hand hygiene campaign. *Am J Infect Control.* Aug 2005;33(6):368-373.

33. Updegraff JA, Emanuel AS, Gallagher KM, Steinman CT. Framing flu prevention--an experimental field test of signs promoting hand hygiene during the 2009-2010 H1N1 pandemic.

Health psychology : official journal of the Division of Health Psychology, American Psychological Association. 2011;30(3):295-299.

34. Grant AM, Hofmann DA. It's not all about me: motivating hand hygiene among health care professionals by focusing on patients. *Psychological science.* 2011;22(12):1494-1499.

35. Bittner MJ, Rich EC, Turner PD, Arnold WH, Jr. Limited impact of sustained simple feedback based on soap and paper towel consumption on the frequency of hand washing in an adult intensive care unit. *Infect Control Hosp Epidemiol.* Mar 2002;23(3):120-126.

36. McGuckin M, Waterman R, Govednik J. Hand hygiene compliance rates in the United States--a one-year multicenter collaboration using product/volume usage measurement and feedback. *Am J Med Qual.* May-Jun 2009;24(3):205-213.

37. Boyce JM. Measuring healthcare worker hand hygiene activity: current practices and emerging technologies. *Infect Control Hosp Epidemiol.* Oct 2011;32(10):1016-1028.

38. Cheng VC, Tai JW, Ho SK, et al. Introduction of an electronic monitoring system for monitoring compliance with Moments 1 and 4 of the WHO "My 5 Moments for Hand Hygiene" methodology. *BMC infectious diseases.* 2011;11(Journal Article):151.

39. Levchenko AI, Boscart VM, Fernie GR. The feasibility of an automated monitoring system to improve nurses' hand hygiene. *International journal of medical informatics.* 2011;80(8):596-603.

40. Armellino D, Hussain E, Schilling ME, et al. Using high-technology to enforce low-technology safety measures: the use of third-party remote video auditing and real-time feedback in healthcare. *Clin Infect Dis.* Jan 1 2012;54(1):1-7.

41. Mayer J, Mooney B, Gundlapalli A, et al. Dissemination and sustainability of a hospital-wide hand hygiene program emphasizing positive reinforcement. *Infection control and hospital epidemiology : the official journal of the Society of Hospital Epidemiologists of America.* 2011;32(1):59-66.

42. das Neves ZC, Tipple AF, Silva e Souza AC, Pereira MS, Melo Dde S, Ferreira LR. Hand hygiene: The impact of incentive strategies on adherence among healthcare workers from a newborn intensive care unit. *Rev Lat Am Enfermagem.* Jul-Aug 2006;14(4):546-552.

43. Erasmus V, Brouwer W, van Beeck EF, et al. A qualitative exploration of reasons for poor hand hygiene among hospital workers: lack of positive role models and of convincing evidence that hand hygiene prevents cross-infection. *Infection control and hospital epidemiology : the official journal of the Society of Hospital Epidemiologists of America.* 2009;30(5):415-419.

44. Schneider J, Moromisato D, Zemetra B, et al. Hand hygiene adherence is influenced by the behavior of role models. *Pediatr Crit Care Med.* May 2009;10(3):360-363.

45. Marra AR, Guastelli LR, de Araujo CM, et al. Positive deviance: a program for sustained improvement in hand hygiene compliance. *Am J Infect Control.* Feb 2011;39(1):1-5.

46. Kretzer EK, Larson EL. Behavioral interventions to improve infection control practices. *American Journal of Infection Control.* 1998;26(3):245-253.

47. Ellingson K, Muder RR, Jain R, et al. Sustained reduction in the clinical incidence of methicillin-resistant Staphylococcus aureus colonization or infection associated with a multifaceted infection control intervention. *Infect Control Hosp Epidemiol.* Jan 2011;32(1):1-8.

48. Pittet D. Improving Compliance with Hand Hygiene. In: Wenzel RP, ed. *Prevention and Control of Nosocomial Infections*. Fourth Edition ed. Philadelphia, PA: Lippincott Williams & Wilkins; 2003.

49. Whitby M, McLaws ML, Slater K, Tong E, Johnson B. Three successful interventions in health care workers that improve compliance with hand hygiene: is sustained replication possible? *American Journal of Infection Control.* 2008;36(5):349-355.

50. Vernaz N, Sax H, Pittet D, Bonnabry P, Schrenzel J, Harbarth S. Temporal effects of antibiotic use and hand rub consumption on the incidence of MRSA and Clostridium difficile. *The Journal of antimicrobial chemotherapy.* 2008;62(3):601-607.

51. Kendall A, Landers T, Kirk J, Young E. Point-of-care hand hygiene: Preventing infection behind the curtain. *Am J Infect Control.* May 2012;40(4 Suppl 1):S3-S10.

52. Eldridge NE, Woods SS, Bonello RS, et al. Using the six sigma process to implement the Centers for Disease Control and Prevention Guideline for Hand Hygiene in 4 intensive care units. *J Gen Intern Med.* 2006;21 Suppl 2(Journal Article):S35-42.

53. Ray AJ, Hoyen CK, Eckstein EC, Donskey CJ. Improving healthcare workers' compliance with hand hygiene: is a picture worth a thousand words? *Infect Control Hosp Epidemiol.* Aug 2002;23(8):418-419.

54. Health Research & Educational Trust. *Hand Hygiene Project: Best Practices from Hospitals Participating in the Joint Commission Center for Transforming Healthcare Project*. Chicago: Health Research & Educational Trust; 2010.

55. Whitby M, Pessoa-Silva CL, McLaws ML, et al. Behavioural considerations for hand hygiene practices: the basic building blocks. *The Journal of hospital infection.* 2007;65(1):1-8.

56. Pittet D. The Lowbury lecture: behaviour in infection control. *J Hosp Infect.* Sep 2004;58(1):1-13.

57. Perlow LA, Gittell JH, Katz N. Contextualizing Patterns of Work Group Interaction: Toward a Nested Theory of Structuration. *Organizational Science.* 2004;15(5):520-536.

58. Champion VL, Sugg Skinner C. The Health Belief Model. In: Glanz K, Rimer BK, Viswanath, eds. *Health Behavior and Health Education: Theory, Research, and Practice*. Fourth Edition ed. San Francisco, CA: Jossey-Bass; 2008.

59. Jenner EA, Jones F, Fletcher BC, Miller L, Scott GM. Hand hygiene posters: selling the message. *The Journal of hospital infection.* 2005;59(2):77-82.

60. Jenner EA, Jones F, Fletcher BC, Miller L, Scott GM. Hand hygiene posters: motivators or mixed messages? *The Journal of hospital infection.* 2005;60(3):218-225.

61. Conzola V, Wogalter MS. A communication-human information processing (C-HIP) approach to warning effectiveness in the workplace. *Journal of Risk Research.* 2001;4(4):309-322.

62. Kim S, Wogalter MS. Habituation, dishabituation, and recovery effects in visual warnings. *Proceedings of the Human Factors and Ergonomics Society 53rd Annual Meeting.* 2009:1612-1616.

63. Wogalter MS, Brelsford JW. Incidental exposure to rotating warnings on alcoholic beverage labels. *Proceedings of the Human Factors and Ergonomics Society 38th Annual Meeting.* 1994:374-378.

64. Wolgalter MS, Laughery KR. WARNING! Sign and label effectiveness. *Current Directions in Psychological Science.* 1996;5(2):33-37.

65. Pittet D, Simon A, Hugonnet S, Pessoa-Silva CL, Sauvan V, Perneger TV. Hand hygiene among physicians: performance, beliefs, and perceptions. *Annals of Internal Medicine.* 2004;141(1):1-8.

66. Hugonnet S, Perneger TV, Pittet D. Alcohol-based handrub improves compliance with hand hygiene in intensive care units. *Archives of Internal Medicine.* 2002;162(9):1037-1043.
67. Perencevich EN, Lautenbach E. Infection prevention and comparative effectiveness research. *JAMA.* 2011;305(14):1482-1483.
68. Platt R, Takvorian SU, Septimus E, et al. Cluster randomized trials in comparative effectiveness research: randomizing hospitals to test methods for prevention of healthcare-associated infections. *Med Care.* Jun 2010;48(6 Suppl):S52-57.
69. Cook TD, Shadish WR, Wong VC. Three Conditions under Which Experiments and Observational Studies Produce Comparable Causal Estimates: New Findings from within-Study Comparisons. *Journal of Policy Analysis and Management.* 2008;27(4):724-750.
70. Patton MQ. *Qualitative research and evaluation methods.* Vol 3rd. Thousand Oaks, CA: Sage Publications; 2002.
71. Ellard DR, Taylor SJ, Parsons S, Thorogood M. The OPERA trial: a protocol for the process evaluation of a randomised trial of an exercise intervention for older people in residential and nursing accommodation. *Trials.* 2011;12:28.
72. Oakley A, Strange V, Bonell C, Allen E, Stephenson J. Process evaluation in randomised controlled trials of complex interventions. *BMJ.* Feb 18 2006;332(7538):413-416.
73. Zwar N, Richmond R, Halcomb E, et al. Quit in general practice: a cluster randomised trial of enhanced in-practice support for smoking cessation. *BMC Fam Pract.* 2010;11:59.
74. Creswell JW, Plano Clark VL. *Designing and Conducting Mixed Methods Research*. Thousand Oaks, CA: Sage Publications; 2007.
75. Rothman AJ, Salovey P. Shaping perceptions to motivate healthy behavior: the role of message framing. *Psychological bulletin.* 1997;121(1):3-19.
76. Ajzen I. The Theory of Planned Behavior. *Organizational Behavior and Human Decision Processes.* 1991;50(Journal Article):179-211.
77. Janz NK, Becker MH. The Health Belief Model: a decade later. *Health education quarterly.* 1984;11(1):1-47.
78. Gould DJ, Chudleigh J, Drey NS, Moralejo D. Measuring handwashing performance in health service audits and research studies. *J Hosp Infect.* Jun 2007;66(2):109-115.
79. Stewardson A, Sax H, Longet-Di Pietro S, Pittet D. Impact of observation and analysis methodology when reporting hand hygiene data. *The Journal of hospital infection.* 2011;77(4):358-359.
80. Sax H, Allegranzi B, Chraiti MN, Boyce J, Larson E, Pittet D. The World Health Organization hand hygiene observation method. *American Journal of Infection Control.* 2009;37(10):827-834.
81. Jadad AR, Moore RA, Carroll D, et al. Assessing the quality of reports of randomized clinical trials: is blinding necessary? *Control Clin Trials.* Feb 1996;17(1):1-12.
82. Chen YC, Sheng WH, Wang JT, et al. Effectiveness and limitations of hand hygiene promotion on decreasing healthcare-associated infections. *PLoS One.* 2011;6(11):e27163.
83. Maury E, Alzieu M, Baudel JL, et al. Availability of an alcohol solution can improve hand disinfection compliance in an intensive care unit. *American journal of respiratory and critical care medicine.* 2000;162(1):324-327.
84. Bischoff WE, Reynolds TM, Sessler CN, Edmond MB, Wenzel RP. Handwashing compliance by health care workers: The impact of introducing an accessible, alcohol-based hand antiseptic. *Archives of Internal Medicine.* 2000;160(7):1017-1021.

85. Haas JP, Larson EL. Impact of wearable alcohol gel dispensers on hand hygiene in an emergency department. *Acad Emerg Med.* 2008;15(4):393-396.
86. Koff MD, Corwin HL, Beach ML, Surgenor SD, Loftus RW. Reduction in ventilator associated pneumonia in a mixed intensive care unit after initiation of a novel hand hygiene program. *J Crit Care.* Oct 2011;26(5):489-495.
87. Pittet D, Sax H, Hugonnet S, Harbarth S. Cost implications of successful hand hygiene promotion. *Infect Control Hosp Epidemiol.* Mar 2004;25(3):264-266.
88. Boscart VM, McGilton KS, Levchenko A, Hufton G, Holliday P, Fernie GR. Acceptability of a wearable hand hygiene device with monitoring capabilities. *J Hosp Infect.* Nov 2008;70(3):216-222.
89. Snyder GM, Thom KA, Furuno JP, et al. Detection of methicillin-resistant Staphylococcus aureus and vancomycin-resistant enterococci on the gowns and gloves of healthcare workers. *Infect Control Hosp Epidemiol.* 2008;29(7):583-589.
90. Shardell M, Harris AD, El-Kamary SS, Furuno JP, Miller RR, Perencevich EN. Statistical analysis and application of quasi experiments to antimicrobial resistance intervention studies. *Clin Infect Dis.* Oct 1 2007;45(7):901-907.
91. *MAXQDA 10 software for qualitative data analysis* [computer program]. Berlin, Germany1998-2012.
92. LeCompte MD, Schensul JJ. *Analyzing and interpreting ethnographic data*. Walnut Creek, CA: AltaMira Press; 1999.
93. Agar MH. *The Professional Stranger: An Informal Introduction to Ethnography*. Second Edition ed. Bingley, UK: Emerald Group Publishing Limited; 2008.
94. Bernard HR. *Social research methods: qualitative and quanitative approaches*. Thousand Oaks, CA: Sage Publications; 2000.
95. Miles MB, Huberman AM. *Qualitative Data Analysis*. Thousand Oaks, CA: Sage Publications; 1994.
