# Supplementary material for: Effect of Frequency of Changing Point-of-Use Reminder Signs on Health Care Worker Hand Hygiene Adherence: A Cluster Randomized Clinical Trial
Source: JAMA Netw Open. 2019 Oct 23;2(10):e1913823. doi: 10.1001/jamanetworkopen.2019.13823 (PMC6820039; doi:10.1001/jamanetworkopen.2019.13823)
Supplement: Supplement 3. — Data Sharing Statement [file jamanetwopen-2-e1913823-s003.pdf]

Data Sharing Statement

Vander Weg. Effect of Frequency of Changing Point-of-Use Reminder Signs on Hand Hygiene Adherence. *JAMA Netw Open*. Published October 23, 2019. 10.1001/jamanetworkopen.2019.13823

Data
Data available: No
